# Mast Cell-Derived SAMD14 Is a Novel Regulator of the Human Prostate Tumor Microenvironment

**DOI:** 10.3390/cancers13061237

**Published:** 2021-03-11

**Authors:** Linda K. H. Teng, Brooke A. Pereira, Shivakumar Keerthikumar, Cheng Huang, Birunthi Niranjan, Sophie N. Lee, Michelle Richards, Ralf B. Schittenhelm, Luc Furic, David L. Goode, Mitchell G. Lawrence, Renea A. Taylor, Stuart J. Ellem, Gail P. Risbridger, Natalie L. Lister

**Affiliations:** 1 Monash Partners Comprehensive Cancer Consortium, Monash Biomedicine Discovery Institute Cancer Program, Prostate Cancer Research Group, Department of Anatomy and Developmental Biology, Monash University, Clayton, Melbourne 3800, Australia; linda.teng@monash.edu (L.K.H.T.); birunthi.niranjan@monash.edu (B.N.); sophie.lee2@monash.edu (S.N.L.); michelle.richards@monash.edu (M.R.); luc.furic@petermac.org (L.F.); mitchell.lawrence@monash.edu (M.G.L.); 2 St Vincent’s Clinical School, Faculty of Medicine, University of New South Wales, Sydney 2010, Australia; b.pereira@garvan.org.au; 3 Kinghorn Cancer Centre, Garvan Institute of Medical Research, Sydney 2010, Australia; 4 Peter MacCallum Cancer Centre, Melbourne 3000, Australia; shivakumar.keerthikumar@petermac.org (S.K.); david.goode@petermac.org (D.L.G.); renea.taylor@monash.edu (R.A.T.); 5 Computational Cancer Biology Program, Peter MacCallum Cancer Centre, Melbourne 3000, Australia; 6 Sir Peter MacCallum Department of Oncology, University of Melbourne, Parkville, Melbourne 3010, Australia; 7 Monash Proteomics and Metabolomics Facility, Department of Biochemistry and Molecular Biology, Biomedicine Discovery Institute, Monash University, Clayton, Melbourne 3800, Australia; cheng.huang@monash.edu (C.H.); ralf.schittenhelm@monash.edu (R.B.S.); 8 Melbourne Urological Research Alliance (MURAL), Monash Biomedicine Discovery Institute Cancer Program, Department of Anatomy and Developmental Biology, Monash University, Clayton, Melbourne 3800, Australia; 9 Monash Partners Comprehensive Cancer Consortium, Monash Biomedicine Discovery Institute Cancer Program, Prostate Cancer Research Group, Department of Physiology, Monash University, Clayton, Melbourne 3800, Australia; 10 School of Health and Wellbeing, University of Southern Queensland, Ipswich 4305, Australia; stuart.ellem@usq.edu.au

**Keywords:** prostate cancer, tumor microenvironment, mast cells, SAMD14, cancer-associated fibroblasts, extracellular matrix

## Abstract

**Simple Summary:**

Mast cells are a type of immune cell that lives within organs and tissues of the body. When tumors develop in these organs, such as in prostate cancer, mast cells secrete multiple factors that can activate the tumor environment and help tumor cells to thrive. Here, we identify a gene called *SAMD14* that is reduced in mast cells obtained from men with prostate cancer. We demonstrate that SAMD14 expression in mast cells can alter their secretions and promote the alignment of matrix fibers that cancer cells use to attach and move around on. By understanding how mast cells regulate their environment, we can reveal new directions of treatment that target the tumor environment as a whole, rather than just the tumor cells themselves.

**Abstract:**

Mast cells (MCs) are important cellular components of the tumor microenvironment and are significantly associated with poor patient outcomes in prostate cancer and other solid cancers. The promotion of tumor progression partly involves heterotypic interactions between MCs and cancer-associated fibroblasts (CAFs), which combine to potentiate a pro-tumor extracellular matrix and promote epithelial cell invasion and migration. Thus far, the interactions between MCs and CAFs remain poorly understood. To identify molecular changes that may alter resident MC function in the prostate tumor microenvironment, we profiled the transcriptome of human prostate MCs isolated from patient-matched non-tumor and tumor-associated regions of fresh radical prostatectomy tissue. Transcriptomic profiling revealed a distinct gene expression profile of MCs isolated from prostate tumor regions, including the downregulation of *SAMD14*, a putative tumor suppressor gene. Proteomic profiling revealed that overexpression of SAMD14 in HMC-1 altered the secretion of proteins associated with immune regulation and extracellular matrix processes. To assess MC biological function within a model of the prostate tumor microenvironment, HMC-1-SAMD14+ conditioned media was added to co-cultures of primary prostatic CAFs and prostate epithelium. HMC-1-SAMD14+ secretions were shown to reduce the deposition and alignment of matrix produced by CAFs and suppress pro-tumorigenic prostate epithelial morphology. Overall, our data present the first profile of human MCs derived from prostate cancer patient specimens and identifies MC-derived SAMD14 as an important mediator of MC phenotype and function within the prostate tumor microenvironment.

## 1. Introduction

Prostate cancer is the second most commonly diagnosed male cancer worldwide, and the fifth leading cause of cancer death in men [1]. Within the prostate gland, normal prostate epithelial development and differentiation are tightly regulated by stromal cells [2,3]. However, following malignant transformation, prostate cancer cells invade the surrounding stroma, activating the tumor microenvironment (TME) [4]. Although it is established that the prostate TME contributes to tumor initiation and disease progression [5,6,7], the reciprocal and heterotypic cellular interactions that occur within the tumor stroma remain less well-defined.

Cancer-associated fibroblasts (CAFs) are present in the early stages of tumorigenesis and differ from their non-malignant prostate fibroblast (NPF) counterparts at the transcriptomic [8], epigenomic [9,10], and proteomic level [11]. Functional assessment of primary prostate fibroblast populations reveals that CAFs retain the ability to initiate and potentiate tumorigenicity in adjacent prostate epithelia [12,13,14,15,16]. In particular, CAFs alter the physical environment of the prostate by depositing an aberrant extracellular matrix (ECM), which can then promote tumor cell invasion and migration [17,18]. Additionally, the reciprocal interactions between CAFs and immune cells modulate the biochemical and physiological structure of the TME to promote or suppress tumor growth [17].

In a study on the global prognostic association of 22 immune cell-types across 14 solid tumors, the presence of mast cells (MCs) was a major predictor of negative patient outcome, including prostate cancer [19]. MCs are unique tissue-resident immune cells that secrete an array of biologically active compounds that can stimulate, modulate, or suppress the TME [20]. Our previous work has demonstrated that mast cells are recruited to the prostate tumor–stromal interface by CAF-derived chemokines [8]. Bioengineered models of the prostatic tumor–stromal interface demonstrate that MCs can secrete factors that modulate the underlying CAF-derived ECM and promote a pro-tumorigenic morphometric transition in adjacent prostate epithelia [8,14].

It is postulated that mast cells acquire distinct molecular and functional abilities within the TME [20], but evidence of the molecular changes which regulate mast cell function remain largely unknown. In this study, we isolated and profiled rare, patient-matched MC populations from human prostate cancer tissue. Our data reveal a novel role for MC-derived SAMD14 in regulating their microenvironment via the remodeling of CAF-derived matrix and promotion of pro-tumor prostate epithelium.

## 2. Results

### 2.1. Mast Cells Isolated from Tumor and Non-Tumorigenic Regions of the Human Prostate Exhibit Distinct Transcriptomic Profiles

Primary MCs were isolated from tumor and non-tumor regions of fresh prostate tissue obtained from a cohort of five men undergoing radical prostatectomy for localized prostate cancer (Figure S1a). Tumors were classified as grade group 2 and 3 (GG2-3) with a median age of 66.9 and a median prostate-specific antigen (PSA) level of 5.8 (Discovery Cohort; Table S1). All patient tissues were validated using immunohistochemistry to confirm tumor versus non-tumor tissue. Tumor samples contained AMACR+ tumor cells and lacked a p63+ basal cell layer, whilst non-tumorigenic prostate glands did not express AMACR and retained a defined p63+ basal cell layer [21] (Figure S1b).

**Figure 1:**
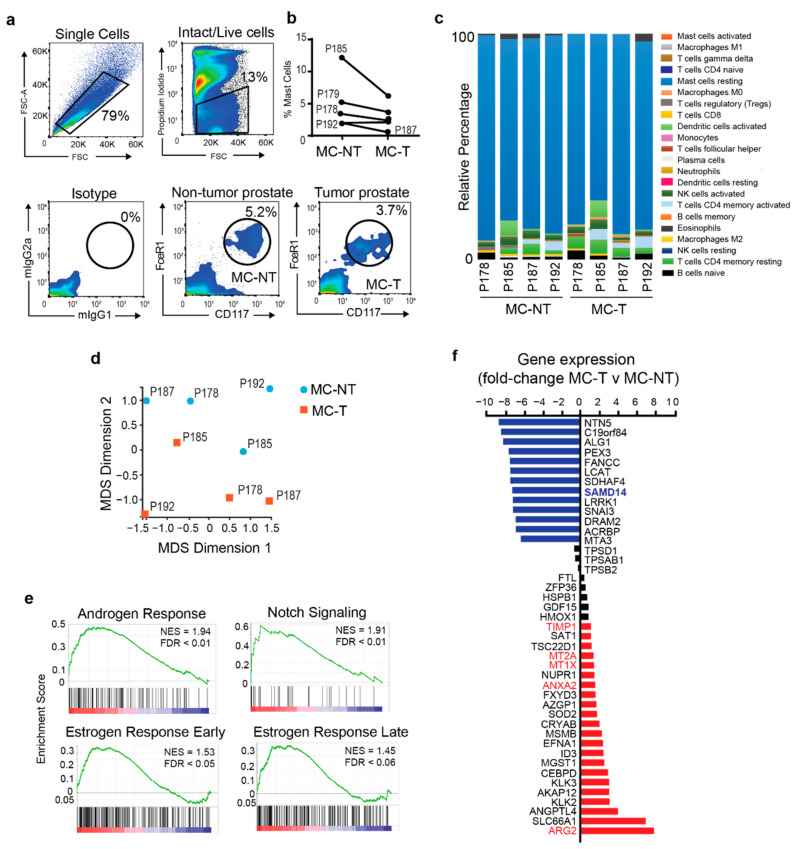
Isolation and transcriptomic profiling of primary mast cells from human prostate tissue (**a**) Flow cytometric plots demonstrate gating strategy for mast cell isolation from primary human radical prostatectomy tissue based on isotype control staining. Plots are representative; n = 5 patients. (**b**) Graph shows the percentage of viable mast cells isolated from paired tumor (MC-T) and non-tumor (MC-NT) patient prostate tissue from 5 localized prostate cancer patients. (**c**) CIBERSORT analysis of primary mast cell RNAseq dataset shows that relative percentage of genes associated with immune subtypes. (**d**) MDS (multidimensional scaling) plot of MC-T (orange squares) and MC-NT (blue circles) patient samples based on gene expression. (**e**) GSEA analysis of MC-T and MC-NT transcriptomes using 50 hallmark gene sets (MSigDB) demonstrates enriched biological pathways in MC-T relative to MC-NT. Normalized enrichment score (NES); false discovery rate (FDR). (**f**) Differential gene expression analysis shows fold-change of genes decreased in MC-T (blue bars) and increased in MC-T (Red bars) based on false discovery rate (FDR) < 0.1 and fold-change (FC) > 2 relative to MC-NT; grey bars indicate FC < 2.

Patient-matched non-tumor and tumor prostate tissues were enzymatically digested to release single cells. MCs were labeled with antibodies towards CD177 and FcεR1 epitopes [22] and isolated via fluorescence-activated cell sorting (FACS) based on isotype control staining (Figure 1a). MCs represented on average 3.2% (±2.3% SD) of viable cells from tumor tissues and 4% (±4.6% SD) of viable cells derived from non-tumor tissues (Figure 1b and Table S2). On average, 6000 (±4.1 × 10^3^ SD) MCs were obtained from prostate tumor tissue and 11,300 (±1.7 × 10^4^ SD) MCs from non-tumor prostate tissue samples (Table S2) and did not significantly differ in total viable MC yield.

RNA sequencing (RNAseq) was used to determine the transcriptomic profile of MCs from non-tumor (MC-NT) and tumor (MC-T) patient prostate tissue. To determine the purity of MCs isolated from human prostate tissue, CIBERSORT analysis was performed on the gene expression dataset. CIBERSORT is an analytical tool developed by Newman et al. (2015) to estimate the relative abundance of immune cell types present in a gene expression dataset [23]. CIBERSORT analysis predicted our MC RNAseq dataset was predominately associated with a resting MC signature, validating the significant enrichment of MCs within our FACS purified prostate samples (Figure 1c).

Multidimensional scaling (MDS) was used to cluster the samples based on their gene expression profile. Despite significant interpatient heterogeneity, our results demonstrate that 3 out of 4 patient MC-T samples cluster away from MC-NT samples through the second dimension, indicating distinct transcriptomic differences between resident MCs populations isolated from discrete prostate microenvironments (Figure 1d). To identify potential regulators of prostate MC function, differential gene expression analysis was used to reveal molecular changes between MC-T and MC-NT patient samples. Gene set enrichment analysis based on the 50 hallmarks dataset (mSigDB) [24] revealed MC-T samples were enriched for multiple biological pathways including, “Androgen Response”, “Notch Signaling”, “Early and Late Estrogen Response” and “Hypoxia” compared to MC-NT (Figure 1e and Table S3). Differential gene expression analysis revealed changes in 35 genes (13 downregulated and 22 upregulated) with an average fold-change > 2 (FDR < 0.1) between MC-T and MC-NT samples (Figure 1f and Table S4). Multiple genes with pro-tumorigenic functions were found to be enriched in MCs isolated from tumor regions, including *ARG2* [25], *ANXA2* [26], metallothioneins (*MT1X* and *MT2A*) [27], and *TIMP1* [28], which are associated with processes such as tumor growth and differentiation, angiogenesis, metastasis, ECM remodeling, and immune escape (Figure 1f; red bar). Tryptase is one of the major proteases secreted by MCs and tryptase+ MCs are reported in both tumor and non-tumor prostate microenvironments [8,14]. A modest reduction (FC < 2; FDR < 0.1) in the expression of tryptase-associated genes (*TPSD1*, *TPSAB1* and *TPSB2*) were reported in MC-T compared to MC-NT (Figure 1f; grey bars). Combined, transcriptomic profiling of MC isolated from primary prostate cancer patients reveals mast cells from tumor regions have a distinct gene expression profile compared to MCs isolated from non-tumor tissues.

One of the top genes identified to be consistently down-regulated in MCs isolated from prostate tumor tissue compared to non-tumor tissue across multiple patients was *SAMD14* (Sterile α-Motif Domain containing protein 14) (Figure 1f and Table S4). SAMD14 belongs to the SAM domain protein family, which exhibits diverse roles and functions including signal transduction and transcriptional repression [29,30]. Limited reports of SAMD14 exist in the literature. However, work by Sun et al. (2008) and more recently, Xu et al. (2020) proposed that epigenetic silencing of *SAMD14* was associated with cancer progression and poor prognosis, leading to the notion of *SAMD14* as a putative tumor suppressor [31,32]. Knockdown of *Samd14* in mice further revealed a role for Samd14 in hematopoietic stem progenitor cell function, including regulation of both myeloid and erythroid progenitor activity [33] and secreted SAMD14 may also function as a B cell autoantigen in primary central nervous system lymphoma [34]. Combined, these observations suggest diverse roles of SAMD14 in multiple cellular contexts. Given the unknown role of SAMD14 in resident prostate MCs we sought to investigate if SAMD14 expression levels could regulate mast cell phenotype and function within the context of the prostate TME.

### 2.2. Overexpression of SAMD14 in HMC-1 Mast Cells Modulates the Secretion of Proteins Associated with Immune Signaling and Regulation of Extracellular Matrix

**Figure 2:**
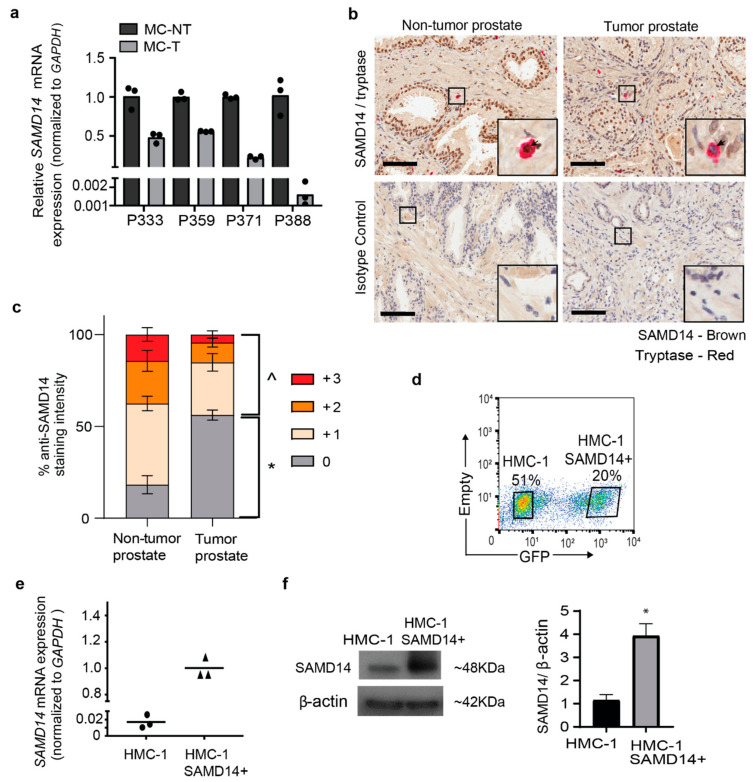
SAMD14 expression in primary mast cells. (**a**) SAMD14 mRNA expression in validation cohort of mast cells isolated from tumor (MC-T) and non-tumor (MC-NT) regions of human prostate tissue (n = 4 patients) normalized to GAPDH. (**b**) Images show representative human prostate tissue sections stained with SAMD14+ (brown) and tryptase+ mast cells (red) and corresponding isotype controls in matched non-tumor and tumor prostate tissues. Scale bars = 100 µm. Images are representative; n = 3 patients. (**c**) Semi-quantitative scoring of SAMD14 staining intensity in tryptase+ mast cells in non-tumor and tumor prostate tissue sections. Bar graph shows the average percentage (±SEM) SAMD14 staining intensity of 3 individual patient tissues (two-way ANOVA Sidak’s multiple comparisons test between tumor and non-tumor prostate tissue regions; ^ *p* < 0.0001 total SAMD14 positivity; * *p* < 0.0001 total SAMD14 negativity). (**d**) Flow cytometric plot shows isolation of live HMC-1-SAMD14 + cell-based GFP expression. Viable cells are gated using propidium iodide. Plot is representative; n = 5. (**e**) SAMD14 mRNA expression in FACS-purified GFP- (HMC-1) and GFP+ (HMC-1-SAMD14+) viable cells normalized to *GAPDH*. (**f**) Western blot show SAMD14 protein expression and β-actin loading control in HMC-1 and HMC-1-SAMD14+ purified cell populations; 25 µg of protein was loaded per lane. Quantification of blot by densitometry shows the average fold-change of SAMD14 expression in HMC-1 and HMC-1-SAMD14+ cells (unpaired student *t*-test; *p* < 0.005). Replicate and uncropped SAMD14 and β-actin western blots are shown in Figure S4.

To validate the reduction of *SAMD14* in MCs isolated from tumor regions (MC-T), we assessed *SAMD14* transcript expression in an independent cohort of patient-matched prostate MCs (n = 4; Validation cohort; Table S1). qPCR confirmed a >50% reduction in *SAMD14* gene expression in MC-T samples compared to MC-NT in 4/4 patients (Figure 2a). Immunohistochemistry demonstrated the expression of SAMD14 within primary human prostate tissue sections, including co-localization with rare, tryptase+ MCs (Figure 2b and Figure S2a). Semi-quantitative analysis of SAMD14+ staining intensity in tryptase+ mast cells across 3 individual patients (Validation cohort; Table S1) revealed a significant reduction in the percentage of SAMD14+ mast cells within prostate tumor sections compared to non-tumor prostate tissue (Figure 2c and Figure S2b). Combined, the data support a reduction in SAMD14 expression at both the transcript and protein level in mast cells within the prostate tumor microenvironment.

When cultured in serum-free-supplemented media, FACS-isolated primary prostate MCs from non-tumor and tumor region of two individual patients gradually declined in cell numbers over prolonged in vitro passage (>100 days) and did not provide sufficient numbers for downstream functional assays (Figure S3a). At present, there is a limited number of immortalized MC lineages available to study mast cell functions. In the absence of a prostate-specific mast cell line, we used the well characterized human mast cell line, HMC-1 to investigate SAMD14 function. HMC-1 cells were originally isolated from a leukemia patient [35,36]. HMC-1 cells have been widely characterized and are capable of modulating their surrounding microenvironment [37]. Importantly, work in our laboratory has revealed HMC-1 are ‘pro-tumorigenic’ when co-cultured with primary prostatic CAFs and promote an invasive matrix within the prostate tumor stromal niche [14].

HMC-1 mast cells were found to express very low levels of *SAMD14* (Figure 2e). Therefore, to assess the biological role of SAMD14 in regulating MC phenotype and fuction, *SAMD14* was overexpressed in HMC-1 cells (HMC-1-SAMD14+) with a construct encoding *SAMD14* and GFP reporter gene. GFP-expressing HMC-1-SAMD14+ cells were isolated via FACS (Figure 2d). GFP-high expressing cells were confirmed to overexpress *SAMD14* at both the transcript (Figure 2e) and protein level (Figure 2f) compared to HMC-1 GFP-negative/low cells. A growth curve was generated and showed no significant differences between the growth of HMC-1 and HMC-1-SAMD14+ cells in culture (Figure S3b).

**Figure 3:**
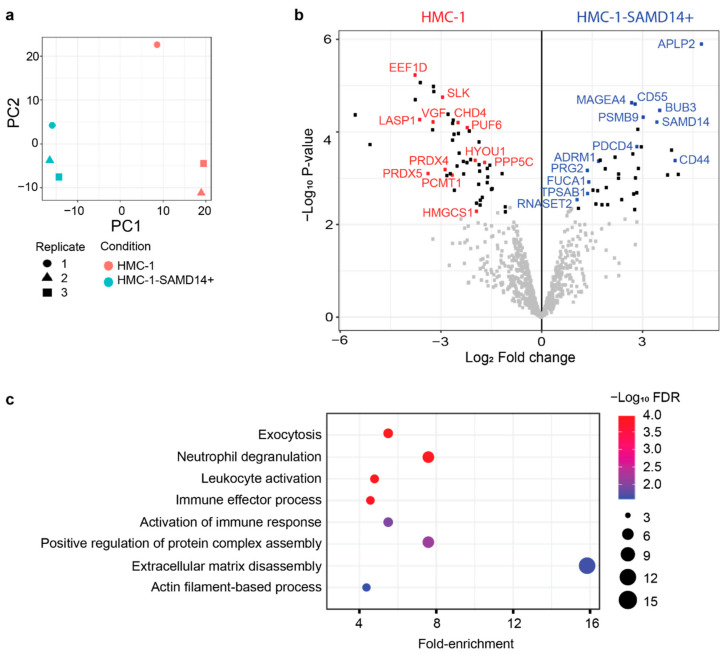
Proteomic analysis of secreted proteins in HMC-1 CM and HMC-1-SAMD14+ CM. (**a**) PCA plot of HMC-1 (red) and HMC-1-SAMD14+ (blue) CM based on secreted protein analysis. For each condition, the experiment was repeated 3 times (circle, triangle, and square). (**b**) Volcano plot visualization of the differentially secreted proteins between HMC-1 (red) and HMC-1-SAMD14+ (blue) based on FDR < 0.05 cutoff (black). (**c**) Functional analysis of the secreted proteins in HMC-1-SAMD14+ CM compared to HMC-1 CM. The plot shows the functional categories that are over-represented in HMC-1-SAMD14+ CM relative to HMC-1 CM using a permutation-based false discovery rate (FDR) analysis.

Mast cells can influence their environment via the secretion of proteins that regulate multiple downstream biological processes and cellular functions [20]. To assess how SAMD14 overexpression may contribute to mast cell phenotype and function, we analyzed the proteins secreted by HMC-1 and HMC-1-SAMD14+ cells in their conditioned media (CM). MC-conditioned media was collected at 48 h from HMC-1 and HMC-1-SAMD14+ cells and analyzed by liquid chromatography-tandem mass spectrometry (LC-MS/MS). A total of 867 proteins were reproducibly quantified in both samples (Table S5). Principal component analysis (PCA) confirmed the stratification of HMC-1 and HMC-1-SAMD14+ secreted proteins (Figure 3a). 148 secreted proteins (63 increased and 84 decreased) were found to be significantly altered (fold-change > 2 and *p* value < 0.05) in HMC-1-SAMD14+ samples compared to HMC-1 controls (Table S6). The distribution of altered proteins was largely balanced between HMC-1 and HMC-1 SAMD14+ samples (Figure 3b) and included alterations in multiple proteins implicated in tumorgenesis and immune regulation (Figure 3b,c). Interestingly, whilst SAMD14 showed nuclear localization by immunohistochemistry in primary human prostate tissue specimens, SAMD14 was also increased in the secretions of HMC-1-SAMD14+ media. It is possible that SAMD14 may regulate intracellular and extracellular functions within the prostate tumor microenvironment. In addition, proteomic analysis revealed enrichment of proteins associated with cellular adhesion, matrix interactions and extracellular matrix remodelling including; APLP2 [38], CD44 [39], ADRM1 [40], RNASET2 [41] and TPSAB1 [42,43] (Figure 3b).

Funtional annotation of proteins with increased abundance in HMC-1-SAMD14+ cell secretions, revealed the enrichment of proteins associated with multiple biological processes including exocytosis, immune regulation, and ECM function (Figure 3c and Table S7). Notably, a 12-fold enrichment of proteins associated with ECM disassembly was reported following SAMD14 overexpression in HMC-1, indicating potential regulation of the prostate tumor microenvironment through interaction with CAFs and extracellular matrix (Figure 3c). Proteins with decreased abundance in HMC-1-SAMD14+ secretions were predominately associated with RNA, metabolic and catabolic processes (Table S8). Collectively, these data indicate that SAMD14 overexpression in MCs can alter the secretion of proteins capable of regulating heterotypic interactions within their surrounding microenvironment, including regulation of immune subsets and extracellular matrix.

### 2.3. SAMD14 Overexpression in Mast Cells Abrogates Extracellular Matrix Alignment in Cancer-Associated Fibroblast

**Figure 4:**
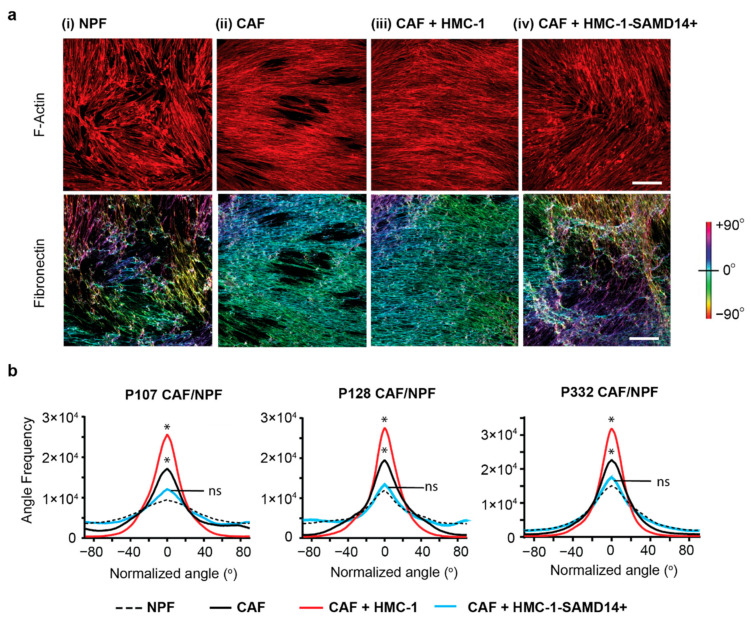
SAMD14 overexpression in mast cells abrogated ECM alignment in CAFs (**a**) Representative image of CAF and NPF derived from P128. Prostate fibroblasts were visualized with F-actin staining (red). Fibronectin staining show ECM fiber alignment for cell-derived matrices produced by (i) NPF, (ii) CAF and (iii) CAF + HMC-1 CM and (iv) CAF + HMC-1-SAMD14+ CM. ECM Images were processed and color-coded to represent the degree of fiber orientation distribution within each sample. Scale bar: 200 µm. (**b**) Quantification of fiber alignment for NPF, CAF and CAF cultured with HMC-1 CM and HMC-1-SAMD14+ CM for the CAF and NPF derived from P332, P107, and P128. Line plots represent analysis with 4 technical replicates per patient, from 4 images per replicate. Statistics performed using Kruskal–Wallis test with Dunn’s post-hoc multiple comparisons test (*, *p* <0.05) to determine statistical significance. Data represented as mean.

Previous work has demonstrated that MCs are able to modulate their stromal microenvironment through interaction with prostate CAFs [8,13,14]. CAFs are one of the most abundant and critical components in the TME [17,18]. They not only secrete ECM proteins that form the structural framework within tissues and organs, they also play a key role in promoting tumorigenesis [17]. Patient-matched pairs of primary prostatic CAF and non-malignant prostatic fibroblast (NPF) were isolated from human prostate tissues as previously reported [44]. To assess whether SAMD14 overexpression in MCs could alter CAF biological functions, conditioned media (CM) containing protein secretions from HMC-1 or HMC-1-SAMD14+ cells were added to CAF/NPF cultures and their extracellular matrix analyzed after 24 h. F-actin was used to visualize CAFs/NPFs, whilst fibronectin staining was used to visualize matrix deposition and orientation (Figure 4a). Matrix images were then color-coded to represent the degree of fiber alignment and orientation as previously reported [45].

NPFs deposit a disorganized matrix, which is reflected by the increase in multicolored fibers that orientate in many different directions (Figure 4a(i)). In contrast, CAFs deposit a highly orientated matrix, which is reflected by a reduction in multicolored fibers and increase in monocolored fibers that depict a strong fiber alignment (Figure 4a(ii)). When HMC-1 secretions were added to CAF cultures, they promoted a more uniform and tightly aligned matrix compared to CAF alone matrix (Figure 4a(iii)). This finding is similar to previous work that indicates HMC-1 mast cells are ‘pro-tumorigenic’ and promote an ‘invasive’ matrix structure when exposed to CAF co-cultures [14]. In contrast, the addition of secretions from HMC-1-SAMD14+ mast cells abrogated this phenotype and instead promoted a disorganized matrix. The loss of fiber orientation and alignment was reflected by an increase in multicolor fibers (Figure 4a(iv)), similar to the matrices produced by NPFs (Figure 4a(i)).

Quantification of matrix fiber angle frequency demonstrated that CAF matrix was significantly more aligned compared to NPF matrix (Figure 4b). Matrix alignment was further enhanced by the addition of HMC-1 CM. In contrast, the addition of HMC-1-SAMD14+ CM reduced fiber alignment, similar to NPF levels. This phenotype was highly reproducible when HMC-1-SAMD14+ secretions were added to three independent pairs of CAF/NPF patient cultures (P107, P128, and P332; Figure 4b).

To investigate the role of SAMD14+ mast cells within the non-malignant prostate microenvironment, HMC-1-SAMD14+/− secretions were added to NPF cultures. HMC-1 CM promoted alignment of NPF-derived matrix and increased fiber orientation similar to CAF-derived matrix (Figure S5a). In contrast, the addition of HMC-1-SAMD14+ secretions to NPF cultures did not promote matrix fiber alignment and orientation, remaining similar to untreated NPF controls (Figure S5a). Quantification of matrix fiber angle frequency in two independent pairs of CAF/NPF patient cultures (P128 and P332) demonstrated that the addition of HMC-1 CM altered NPF-matrix to a more CAF-like matrix (Figure S5b). In contrast, these pro-tumorigenic functions were abrogated following the overexpression of SAMD14 in HMC-1 cells, where matrix alignment remained similar to untreated NPF control levels (Figure S5b). Together, these results support the role of SAMD14+ mast cells in promoting a ‘normalized’ extracellular matrix reflective of the non-tumor prostate microenvironment.

### 2.4. SAMD14-Overexpressing Mast Cells Reduce the Tumorigenic Phenotype of Prostate Epithelium

Changes in the underlying matrix produced by CAF have been shown to directly alter the tumorigenic phenotype and invasive potential of tumor epithelium [17]. Previous work has shown that non-malignant prostate epithelial cells (BPH-1) become pro-tumorigenic when exposed to CAF, but not NPF, co-cultures both in vitro and in vivo [12,13,14]. Pro-tumorigenic epithelium is further enhanced upon addition of HMC-1 to CAF cultures, where the MCs act to promote an invasive CAF matrix and indirectly increase epithelial tumorigenicity [14].

**Figure 5:**
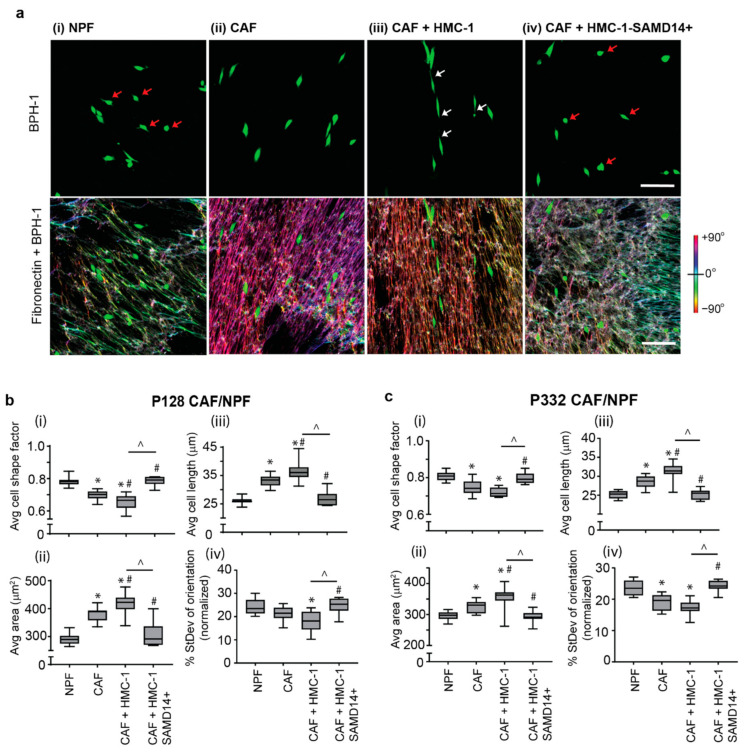
SAMD14 overexpression in mast cells reduces pro-tumor prostate epithelial morphology. (**a**) Representative images show BPH-1 cell morphology when cultured with fibroblasts (CAF/NPF) derived from P128. Corresponding fibronectin staining after image processing to represent the degree of ECM fiber alignment produced by (i) NPF (from left), (ii) CAF, (iii) CAF + HMC-1 and (iv) CAF + HMC-1-SAMD14+ CM. Scale bar: 100 µm. (**b**) Quantification of (i) shape factor, (ii) area, (iii) cell length and (iv) standard deviation of orientation of BPH-1 cells cultured on NPF, CAF, CAF + HMC-1 CM and CAF + HMC-1-SAMD14+ CM (P128) after 24 h of co-culture. Box and whisker plots represent the max to min value of BPH-1 shape factor, area, cell length and standard deviation of orientation. (**c**) Quantification of (i) shape factor, (ii) area, (iii) cell length and (iv) standard deviation of orientation of BPH-1 cells on NPF, CAF, CAF + HMC-1 CM and CAF + HMC-1-SAMD14+ CM (P332) after 24 h of co-culture. Box and whisker plots represent the max to min value of BPH-1 shape factor, area, cell length and standard deviation of orientation. Graphs represent analysis of 3 images per replicate and 4 replicates are conducted per patient (>50 BPH-1 cells/image). Statistics performed using two-way ANOVA with Tukey’s post hoc test for average shape factor, area and cell length (* = *p* < 0.0001 compared to NPF; # = *p* < 0.005 compared to CAF; ^ = *p* < 0.0001 compared to CAF+ HMC-1) and a one-Way ANOVA with Tukey’s post hoc for average standard deviation of orientation (* = *p* < 0.001 compared to NPF; # = *p* < 0.05 compared to CAF; ^ = *p* < 0.001 compared to CAF+ HMC-1).

To assess whether SAMD14 overexpression in HMC-1 cells could alter prostate epithelial tumor phenotype, fluorescently-labelled BPH-1 prostate epithelial cells co-cultured with NPF or CAF or CAF supplemented with HMC-1 or HMC-1-SAMD14+ conditioned media over a period of 24 h (Figure 5a). The morphological transition of prostate epithelia was quantified by measuring changes in BPH-1 cellular shape, length, and orientation through confocal microscopy and 3D morphometric analysis to provide an objective measurement of phenotypic transition [13,14].

When cultured on an NPF matrix, BPH-1 cells remained rounded in shape and were randomly orientated with fewer cellular protrusions (Figure 5a(i); red arrows). In contrast, the addition of BPH-1 to CAF matrix resulted in a pro-tumorigenic epithelial phenotype, with BPH-1 cells becoming elongated and more spindle-like, and were highly aligned along the CAF-ECM fibers (Figure 5a(ii)). The addition of HMC-1 CM to the CAF cultures further enhanced the malignant phenotype of prostate epithelial cells, which were highly orientated with the underlying ECM (Figure 5a(iii); white arrows). In contrast, the addition of CM from HMC-1-SAMD14+ cells reduced tumor epithelial morphology. BPH-1 cells were round and smaller in shape (Figure 5a(iv); red arrow), were randomly orientated and had fewer protrusions similar to phenotypic presentation observed when BPH-1 are co-cultured with NPFs (Figure 5a(i)).

Quantitation of morphological cell parameters showed that when cultured with CAF, BPH-1 cells decreased their shape factor, increased their average area, became more elongated, and were more aligned compared to when BPH-1 cultured on NPF (Figure 5b,c). These features were significantly enhanced upon the addition of HMC-1, but not HMC-1-SAMD14+ conditioned media. Indeed, HMC-1-SAMD14+ media could abrogate BPH-1 pro-tumor phenotype, similar to levels observed when BPH-1 were cultured with NPF. These results were highly reproducible when BPH-1 were cultured with two separate pairs of CAF/NPF patient primary cultures (P128—Figure 5b and P332—Figure 5c). When HMC-1 CM was added to NPF + BPH-1 co-cultures, BPH-1 cells became elongated and aligned with the ECM fibers, similar to the morphological changes observed when BPH-1 cells were grown on CAF matrices (Figure S5a). In contrast, the addition of HMC-1-SAMD14+ CM abrogated these properties and did not significantly alter BPH-1 morphology in NPF co-cultures (Figure S5a). Quantification of BPH-1 morphological cell parameters when cultured with two individual patient-derived NPF lines (P128 and P332) showed that the addition of HMC-1 CM, but not HMC-1-SAMD14+ CM, promoted the pro-tumorigenic morphologic transition of BPH-1 prostate epithelia. (Figure S5c).

Importantly, the addition of HMC-1-SAMD14+/− CM to BPH-1 cells directly did not significantly alter their morphology (Figure S6), suggesting that CAF/NPF-derived matrices were required to mediate alterations in BPH-1 morphology. To further determine if the morphological changes occurring in BPH-1 were due to alterations in the underlying CAF-derived matrix, as opposed to a direct effect on BPH-1 cells, washout assays were performed with HMC-1-SAMD14+/− CM. CAF/NPF cultures were treated with HMC-1 or HMC-1-SAMD14+ CM for 24 h, before media was removed and cultures washed prior to the seeding of BPH-1 cells on CAF/NPF matrices (Figure S7). In this set of experiments, CAFs maintained their enhanced fiber alignment, indicating maintenance of pro-tumorigenic matrix despite removal of HMC-1 CM. Notably, BPH-1 cells maintained their enhanced morphological changes when CAFs were pre-exposed to HMC-1 CM, but not HMC-1-SAMD14+ CM, in line with previous observations (Figure S7a). Quantification of ECM fiber alignment (Figure S7b) and BPH-1 morphology (Figure S7c) was consistent across two independent CAF/NPF patient lines. Combined, these data indicate that SAMD14 overexpression in mast cells acts to regulate mast cell phenotype and function within the prostate microenvironment, acting on fibroblasts to normalize the deposition and alignment of matrix and regulate prostate epithelial morphology.

## 3. Discussion

Infiltrating immune cells in solid tumors actively participate in tumorigenesis and can significantly influence the course and progression of malignant disease. Mast cells are granulocytic immune cells of myeloid origin that play an active role in shaping the microenvironment to favor malignant transformation and invasion of adjacent epithelial cells [8,14]. Previous work by our group and others has shown that mast cells are a resident prostatic stromal cell population [46], which are increasingly recruited to the tumor interface during malignancy [8,14,47,48]. From here, mast cells can modulate the TME, leading to tissue remodeling, fibrosis, angiogenesis, lymphangiogenesis, as well as activation of the innate and adaptive immune system [20]. It is postulated that mast cells acquire distinct molecular and functional abilities within the tumor microenvironment [20]. However, in prostate cancer, the specific mechanisms by which mast cells modulate these interactions is still not well understood. To further elucidate prostate cancer-specific mast cell phenotypes and functions, we isolate and profile primary mast cells from matched tumor and non-tumor regions of prostate tissue obtained from localized prostate cancer patients. Our data reveal that, although a similar percentage of mast cells was observed between tumor and non-tumor regions of the prostate, there were significant alterations in their transcriptional profile and indicate that mast cell phenotype and function may be dependent on their specific localization within the prostate. Here, our study demonstrates that SAMD14 acts to regulate prostate mast cell biological functions through the normalization of extracellular matrix in prostate fibroblasts which can impede prostate epithelial tumor morphology.

Mast cells are derived from hematopoietic stem cells in the bone marrow, and are a highly heterogeneous, plastic population of immune cells [49]. In vivo, mast cell progenitors are released into the blood where they migrate to peripheral tissues [50]. Only when they are localized within these tissues and in the presence of stem cell factor (SCF) do they terminally differentiate and mature [50,51]. As a result of these characteristics, mast cell research has been hampered by difficulties in successfully isolating and expanding primary mast cells in culture. Furthermore, as mast cells are highly influenced by their microenvironment, their morphology, granule content, surface receptor expression, and functionality is principally based on their tissue micro-location [20]. This results in a spectrum of mast cell phenotypes across different tissues and even at different locations within the same tissue, as seen in the lung [52].

Using low numbers of mast cells isolated from primary prostate tissue, we were able to reveal distinct transcriptomic differences between MCs isolated from different prostate microenvironments across multiple primary prostate specimens. Mast cells from tumor regions were enriched for Androgen and Estrogen Response pathways compared to MCs isolated from non-tumor prostate tissues. It has previously been established that MCs are hormone-responsive, and express both estrogen and androgen receptors [53,54,55]. Moreover, we have previously shown that mast cell infiltration into murine prostate tissue is enhanced with elevated endogenous estrogen levels and may regulate chronic inflammation associated with prostatitis and pre-malignant lesions [56]. Thus, the alteration of hormone-driven pathways in resident MCs may contribute to their pro-tumorigenic function, particularly in the context of hormone-dependent cancers such as prostate cancer. Although our study focused on treatment-naïve prostate cancer patients, mast cell phenotype and function may be further altered in patients who receive hormonal therapies, such as androgen-deprivation therapy (ADT), and influence disease progression.

In this study, we show that overexpression of SAMD14 is sufficient to alter MC phenotype and function. Previously, Samd14 has been shown to regulate signaling pathways critical for hematopoietic stem/progenitor cell survival and function in murine myeloid and erythroid lineages [33]. Our data presented herein further supports a role for SAMD14 in regulating the function of myeloid-derived resident populations of prostate MCs, highly dependent on their discreet microenvironmental milieu. Indeed, our proteomic analysis reveals SAMD14 overexpression in MCs alters their secreted profile to enrich for proteins associated with both immune regulation and ECM function, indicating that MCs may influence multiple cellular compartments within the TME.

CAFs are one of the most abundant and critical cellular components involved in promoting tumor development and progression [57]. During early tumor initiation, CAFs are activated by adjacent tumor cells via paracrine signaling to cultivate a desmoplastic, pro-tumorigenic microenvironment [58]. In vivo, CAFs secrete abundant, aberrant ECM [11] and also recruit immune cells to the tumor site, including MCs [8,14]. Previously, our group has shown that addition of MC secretions to a bioengineered prostate microenvironment promoted the tumorigenicity of prostate epithelium via alterations to the underlying extracellular matrix structure produced by CAFs [14].

In this study, we demonstrate that SAMD14 alters the secreted profile of MCs to promote the normalization of ECM matrix deposited by prostatic CAFs. Using an in vitro co-culture model that reflects stromal/tumor interactions within the prostate microenvironment, we further show that HMC-1-SAMD14+ mast cells act directly on CAF to alter their secreted matrix and indirectly regulate changes in the tumorigenic phenotype of adjacent prostate epithelium. These functional data are supported by proteomic profiling, where overexpression of SAMD14 in HMC-1 cells resulted in the significant enrichment of proteins related to “Extracellular matrix disassembly” and “Actin filament-based process”, indicating SAMD14+ MCs may play a role in regulating ECM organization and cytoskeletal changes to elicit functional changes within the prostate microenvironment.

ECM remodeling can occur through multiple mechanisms, including aberrant ECM deposition, proteolytic ECM degradation, chemical modification as well as force-mediated ECM remodeling triggered through integrin-ECM signaling [59]. Following SAMD14 overexpression, proteomic analysis revealed alterations in a number of proteins secreted by mast-cells that are associated with matrix interaction and remodeling. TPSAB1 (Tryptase), is a major mast cell secretory protease, which has been shown to cleave, activate, or degrade ECM components and mediate ECM remodeling within the tumor microenvironment [42,43]. In addition, CD44 is a cell-adhesion receptor specific for ECM constituent Hyaluronan (HA) and also exhibits affinity for fibronectin and collagen matrix molecules [39]. Soluble CD44 is secreted by multiple cell types including MCs [60,61], and has been shown to complex with cellular matrix components to regulate ECM organization and remodeling and influence cell-matrix interactions [62]. It is possible that individual or a combination of proteins regulated by SAMD14+ mast cells may contribute to the alteration of CAF-derived matrix phenotype and function reported in this study. Further investigation into the regulation of CAF matrix by SAMD14+ MC secreted proteins will provide important insight into the prostate tumor microenvironment and disease progression.

## 4. Materials and Methods

### 4.1. Human Patient Sample Collection

Whole human radical prostatectomy tissue was collected with consent from patients at Epworth HealthCare and Cabrini Hospital according to human ethics approvals obtained from Cabrini Hospital (Monash Health RES-20-0000-107C and Monash Health RES-20-0000-103C), Epworth HealthCare (Monash Health RES-19-0000-407E), and Monash University (1636). The pathology of prostate tissue was confirmed by a board-certified pathologist (TISSUPATH) as previously described [63]. Briefly, the whole surface of the prostate was inked to identify surgical margins. Tumor regions were located based on biopsy histopathology reports and palpation. Two to three pieces of tissue (~1 × 4 mm in size) were dissected from the tumor area, and tumor content was confirmed through rapid H&E staining of frozen sections. Once the tumor and non-tumor regions were identified, ~500 mg of fresh tissue was dissected and transported to Monash University laboratories on ice in transport medium (RPMI 1640 (School of Biomedical Sciences, Media and Prep Services, Monash University) supplemented with phenol red, 10% heat-inactivated HyClone fetal bovine serum (HI-FBS; GE Healthcare) and 100 U/mL penicillin and 100 mg/mL streptomycin (P/S; Sigma-Aldrich), 0.5 µg/mL amphotericin B antimycin (Life Technologies) and 100 µg/mL gentamicin (Life Technologies)). All fresh prostate tissue used in this study was processed within two hours following surgery. Representative tissue pieces were obtained from each patient specimen for validation of non-tumor and tumor content via immunohistochemistry (refer Section 4.2).

### 4.2. Prostate Tissue Fixation and Immunohistochemistry

Prostate tissue specimens were processed and embedded in paraffin wax and serially sectioned at 5 μm for histological and/or stereological analysis. Immunohistochemistry was performed using the Leica BOND-MAX^TM^ automated system (Leica Microsystems, Australia), according to manufacturer’s instructions. The automated procedure consisted of: Dewaxing of tissue sections, blocking endogenous peroxidase activity using 0.3% H_2_O_2_ in methanol, heat-induced antigen retrieval (pH 9.0, 30 min), incubation with primary antibodies for 15–60 mins, incubation with a peroxidase-labelled polymer for 30 min and a subsequent incubation with a substrate-chromogen (DAB or Fast red) for 10–15 mins. Nuclear counterstaining was performed with hematoxylin.

To validate prostate tissue pathology, dual antibody staining was performed on representative tissue pieces using antibodies against AMACR (DAKO) and p63 (Leica Microsystem). Tumor content was defined as AMACR+ tumor cells in the absence of p63+ basal cell layer, whilst non-tumor tissue lacked AMACR+ cells and maintained p63+ glandular structures [21].

To quantify SAMD14+ protein expression in primary prostate mast cells, dual immunohistochemistry was performed on tumor and non-tumor prostate tissue sections using antibodies against SAMD14 (Novus Biologicals) and tryptase (Leica Microsystem) or isotype control antibodies (Table S9). Slides were imaged using an Aperio ScanScope AT Turbo slide scanner (Leica Microsystems). Semi-quantitative scoring of SAMD14 staining intensity was manually assessed using ImageScope analysis software (Aperio). Mast cells were identified within prostate tissue based on positive cytoplasmic staining for tryptase [64] and nuclear SAMD14 staining intensity was scored based on a scale of increasing intensity from 0 to +3. At least 40 mast cells were identified and scored from each patient tissue. Data are presented as a percent of the total number of mast cells within patient tissue (n = 3 patients). Antibodies used for immunohistochemistry and staining conditions are detailed in Table S9.

### 4.3. Fluorescence-Activated Cell Sorting of Primary Mast Cells

Non-Tumor and Tumor Prostate tissue collected were digested in RPMI-1640 containing 1 U/mL Liberase^TM^ (Roche) supplemented with 0.02% (*w*/*v*) DNase-I (Roche) for 3–5 h at 37 °C in a rotating oven. Digested prostate cells were incubated with anti-human FcεR1-APC (clone AER-37; eBioscience^TM^) and anti-human CD117-PE (Clone 104D2; BD Biosciences) or anti-mouse IgG_2b_-APC (clone eBMG2b; eBioscience^TM^) and anti-mouse IgG_1_-PE (clone P3.6.2.8.1; eBioscience^TM^) for 1 h at 4 °C in the dark as described in Radinger et al. (2010) [22]. 100 ng/mL of propidium iodide (Sigma-Aldrich) was used to exclude dead cells. Primary mast cells were isolated based on isotype control staining. Sorting of primary mast cells was performed on a BD Influx cell sorter (BD Biosciences) using a 100 μM nozzle at 20 psi. Cell fractions were analyzed with Sortware (BD Biosciences). Prostate mast cells were isolated from the double positive fraction (FcεR1+ and CD117+) compared to isotype control staining. Prostate mast cells obtained were lysed in lysis buffer supplied by RNeasy Micro kit (QIAGEN).

### 4.4. RNAsequencing and Analysis

Total RNA from prostate mast cells was isolated using RNeasy Micro Kit (QIAGEN) with an on-column DNase-I treatment according to the manufacturer’s instruction. Total RNA was quantified in a Nanodrop ND-1000 spectrophotometer, checked for purity and integrity in a Bioanalyzer-2100 device (Agilent Technologies). RNA was amplified with Clontech SMART-Seq V4 Ultra-Low Input RNA kit (Clontech) according to manufacturer’s protocol. The amplified RNA were submitted to Medical Genomics Facility in Monash Health Translation Precinct (Clayton, Australia) for RNA sequencing. Libraries were prepared by Ovation Ultralow System V2 using Nugen protocol M01379v1 (NuGEN, San Carlos, CA, USA). C-bot clustering was generated using 12 pM of library pool with illumina Protocol 15006162 v02 (Illumina, San Diego, CA, USA). 50 bp Single-End Reads sequencing was done with HiSeq1500 High Output platform (Illumina, San Diego, CA, USA). For each sample, 2 lanes of 150 M raw reads per lane of data were obtained for downstream analysis. MCs isolated from patient 179 (P179) failed RNAsequencing and were omitted from this study.

Raw reads in Fastq files were analyzed with RNAsik pipeline [65], producing genes count matrix together with QC metrics, summarized in MultiQC report. RNAsik was set to run STAR aligner option [66] and featureCounts for genes quantification [67]. Human reference files, GTF and FASTA, were downloaded from the Ensembl database, version GRCh38, release 83. Differential gene expression was analyzed using Degust [68] web tool and limma voom [69] was selected for differential expression analysis. Degust [68] largely follows limma voom workflow with typical counts per million (CPM) library size and trimmed mean of M values (TMM) [70] conducted for the normalization of the RNA composition. Principal Component Analysis or Multidimensional scaling were performed in Degust [68]. Gene Set Enrichment Analysis (GSEA) against 50 cancer hallmarks pathway (MSigDB v7.2) were conducted using a pre-ranked GSEA method with the gene list ranked by LogFC [24,71].

### 4.5. CIBERSORT

CIBERSORT is an analytical tool which accurately quantifies the relative levels of distinct immune cell types within a complex gene expression mixture [23]. To characterize and to quantify each immune cell subtype, CIBERSORT uses gene expression signatures consistent of ~500 genes and considered a minimal representation for each cell type based on those values. Here, we have applied the original CIBERSORT gene signature file LM22, which defines 22 immune cell subtypes, and used it to analyzed the primary mast cells RNAseq dataset. The data are run using the default signature matrix at 100 permutations.

### 4.6. Quantitative RT-PCR

Total RNA from primary mast cells was isolated using RNeasy Kit (QIAGEN) with an on-column DNase-I treatment. Total RNA was amplified REPLIG WTA single cell kit (QIAGEN) according to the manufacturer’s instructions. Gene expression was examined by Real-Time qPCR performed on samples using Power SYBR^TM^ Green Master Mix (Thermo Fisher Scientific) and Mx3000 real-time QPCR Software (Agilent Technologies). The relative mRNA expression levels of SAMD14 (F′-CGAGAACCCGTGGATGAAGT-3′; R′-CGGAGGATCCAGGCAGAAAG-3′) was calculated using the ΔΔCt method and normalized against GAPDH (F′-ACCACCAACTGCTTAGCACC-3′; F′-CCATCCACAGTCTTCTGGGT-3′) reference gene expression.

### 4.7. Primary Mast Cells Culture

Flow sorted primary mast cells were maintained in human mast cell culture media (StemPro^TM^-34 Serum-Free media (Gibco), StemPro-34 Nutrient Supplement (Gibco), L-Glutamine (2 mM) (Gibco), Penicillin (100 U/mL)/Streptomycin (100 µg/mL) (Gibco), recombinant human stem cell factor (100 ng/mL) (PeproTech), recombinant human Interleukin 6 (100 ng/mL) (PeproTech) and Recombinant human Interleukin-3 (30 ng/mL) (PeproTech)) at 37 °C in 5% CO_2_, with media changes every 5–7 days [22]. Primary mast cell numbers were determined with TC-20^TM^ Automated Cell Counter system (Bio-Rad) according to manufacturer’s protocol at every media change. Growth curves for primary mast cells are shown in Figure S3a.

### 4.8. Cell Lines

The non-malignant prostate epithelial cell line, BPH-1 [72] (kindly provided by Dr. Simon W. Hayward, Vanderbilt University, USA) was maintained in RPMI 1640 (School of Biomedical Sciences, Media and Prep Services, Monash University) supplemented with phenol red, 5% heat-inactivated HyClone fetal bovine serum (HI-FBS; GE Healthcare) and 100 U/mL penicillin and 100 mg/mL streptomycin (P/S; Sigma-Aldrich)) at 37 °C in 5% CO_2_. Media for BPH-1 was changed every 2–3 days. Human mast cell line, HMC-1 cells [36] (kindly provided by Dr. Joseph Butterfield; Mayo Clinic, USA) were maintained in HMC-1 media (Iscove’s modified Dulbecco’s medium with L-glutamine (IMDM; Gibco), 1.2 mM α-thioglycerol (Sigma-Aldrich), 10% HI-FBS and P/S) at 37 °C in 5% CO_2_, with media changes every 5–7 days.

### 4.9. Transfecting of SAMD14 into HMC-1 Cell Line

A custom-designed plasmid for SAMD14 (NM_174920.3) cloned into pcDNA3.1(+)-C-eGFP vector which confers neomycin resistance to allow stable generation of transgenic cell-line and a GFP reporter gene for isolation of SAMD14 expressing cells was constructed according to manufacturer’s instruction (GenScript). The plasmid was transfected into HMC-1 cells with Lipofectamine^TM^ 3000 according to manufacturer’s protocol (Thermo Fisher Scientific). Briefly, 5 × 10^5^ HMC-1 cells were seeded into each well of the 6 well dish. The suspension cells were cultured in 2 mL of OptiMEM reduced serum media (Thermo Fisher Scientific) for 2 h prior to transfection. 15 ug SAMD14 pcDNA3.1(+)-C-eGFP plasmid were mixed with 22.5 µL of Lipofectamine 3000 reagent and 30 µL of P3000^TM^ reagent in 1.5 mL OptiMEM reduced serum media. After 15 min incubation at room temperature, 250 µL the mixture was added into each well. After 24 h transfection, HMC-1 transfected cells were washed with phosphate-buffered saline. and the cells were resuspended in fresh HMC-1 media. After 48–72 h, HMC-1 SAMD14+ GFP expressing cells were sorted using flow cytometry. The sorted cells were maintained in HMC-1 media supplemented with 500 µg/mL of G418 antibiotic (Thermo Fisher Scientific).

### 4.10. Western Blot

HMC-1 and HMC-1 SAMD14 cells were washed in phosphate-buffered saline (School of Biomedical Sciences, Media and Prep Services, Monash University) and lysed in 200 µL of radioimmune precipitation assay buffer (RIPA; Milli-pore) supplemented with Roche Complete^TM^ protease inhibitor cocktail (Roche). Protein concentration of the lysed cells were determined using a reducing agent and detergent compatible (RC DC) protein assay kit (Bio-Rad). 25 µg of proteins of each sample were loaded into each well and separated by standard SDS-PAGE. Proteins were transferred onto PVDF membranes (Millipore, Minneapolis, MN) in transfer buffer (6 g/L tris base, 3 g/L glycine, 0.36 g/L SDS, 20% methanol) at 100 V for 1 h. Membranes were blocked in blocking solution (5% (*w*/*v*) skimmed milk powder in 0.05% (*v*/*v*) Tween 20 (TBST) blocking solution). Membranes were either probed with antibodies specific for SAMD14 (NBP2-13278; Novus Biologicals; 1:1000) overnight at 4 °C or β-Actin (A5316; Sigma; 1:100,000) for 1 h at room temperature. After washing, the blots were incubated with either polyclonal goat α-rabbit immunoglobulins/HRP (Dako; 1:10,000) or polyclonal goat α-mouse immunoglobulins/HRP (Dako; 1:10,000) for 30 min at room temperature. Immunoreactive bands were detected on medical X-ray films (Agfa HealthCare) using Clarity Western ECL Substrate (Bio-rad). Densitometric analysis of immunoreactive protein bands were quantitated using ImageJ (NIH) and calculated as units = Intensity/mm^2^. After normalizing the levels with β-actin for each sample, semi-quantitative results for SAMD14 protein expression were calculated. Uncropped western blots are shown in Figure S4.

### 4.11. Mass Spectrometry

HMC-1 and HMC-1-SAMD14+ cells were seeded at 3 × 10^5^ cells/mL and cultured in Iscove’s modified Dulbecco’s medium (IMDM; Gibco) without serum for 48 h. Cells were centrifuged at 1000 rpm for 5 min. The supernatant from this culture was then collected and filtered with a 0.22 μm filter. Each experiment was conducted 3 times. Equal amounts of conditioned media (5 mL) was concentrated and buffer exchanged with Amicon Ultra 4 centrifugal Filter Units (Merck). The protein concentration was determined with Bicinchoninic Acid assay (BCA, Thermo Fisher Scientific). The protein was reduced, alkylated, and trypsin-digested overnight. The digested peptides were cleaned up with SDB-RPS StageTips (3 M).

Using a Dionex UltiMate 3000 RSLCnano system equipped with a Dionex UltiMate 3000 RS autosampler, an Acclaim PepMap RSLC analytical column (75 µm × 50 cm, nanoViper, C18, 2 µm, 100 Å; Thermo Fisher Scientific) and an Acclaim PepMap 100 trap column (100 µm × 2 cm, nanoViper, C18, 5 µm, 100 Å; Thermo Fisher Scientific), the tryptic peptides were separated by increasing concentrations of 80% acetonitrile ACN/0.1% formic acid at a flow of 250 nL/min for 120 min and analyzed with a QExactive HF mass spectrometer (Thermo Fisher Scientific). The instrument was operated in the data-dependent acquisition mode to automatically switch between full scan MS and MS/MS acquisition. Each survey full scan (*m*/*z* 375–1575) was acquired in the Orbitrap with 60,000 resolution (at *m*/*z* 200) after accumulation of ions to a 3 × 10^6^ target value with maximum injection time of 54 ms. Dynamic exclusion was set to 15 s. The 12 most intense multiply charged ions (z ≥ 2) were sequentially isolated and fragmented in the collision cell by higher-energy collisional dissociation (HCD) with a fixed injection time of 54 ms, 30,000 resolution and automatic gain control (AGC) target of 2 × 10^5^.

The raw data files were analyzed with the MaxQuant software suite v1.6.5.0 [73] and its implemented Andromeda search engine [74] to obtain protein identifications and their respective label-free quantification (LFQ) values using standard parameters. The proteomics data were further analyzed using LFQ-Analyst [75].

The mass spectrometry proteomics data have been deposited to the ProteomeXchange Consortium via the PRIDE partner repository with the dataset identifier PXD022782 [76].

### 4.12. Proteomic Functional Annotation Analysis

The significantly regulated proteins from secretome analysis were submitted to STRING online database for protein-protein interaction (PPI) and functional enrichment analysis, including Gene Ontology, KEGG, and Reactome pathways [77]. Only when the functional categories have a fold enrichment > 2 and FDR < 0.05, with an interaction score > 0.4 were considered and plotted in R [78] using package ggplot2 [79].

### 4.13. Isolation of Primary Prostatic Fibroblasts (CAFs and NPFs)

Tissue was collected from radical prostatectomy (RP) specimens for CAFs and NPFs as previously described [13,44]. Briefly, CAFs and NPFs were then isolated from tumor and non-tumor regions identified and excised by a trained pathologist. Whole tissue was enzymatically digested to release single cells and cultured in fibroblast media RPMI 1640 (School of Biomedical Sciences, Media and Prep Services, Monash University) supplemented with phenol red, 5% heat-inactivated HyClone fetal bovine serum (HI-FBS; GE Healthcare), 1 nM testosterone (Sigma-Aldrich), 10 ng/mL basic fibroblast growth factor (bFGF; PeproTech), 100 U/mL penicillin and 100 mg/mL streptomycin (P/S; Sigma-Aldrich). Cells were maintained at 37 °C in 5% CO_2_, 5% O_2_ atmosphere, with media changes every 2–3 days. Matched fibroblasts from 3 patients were used for this study. Details of the patients’ information is in Table S10. The matched fibroblasts from these 3 patients have previously been characterized, authenticated, and published [9,14].

### 4.14. In-Vitro Cellularized Co-Culture Assay

An in-vitro cellularized co-culture model was used as previously described [13,14]. Briefly, patient-matched primary CAFs or NPFs were seeded in 48 well plates at a concentration of 1.0 × 10^4^ cells/mL and cultured for 8–10 days in fibroblast media to yield a dense monolayer of extensive ECM deposition. Then BPH-1 pre-stained with CellTracker Green CMFDA (CtC; Invitrogen) were seeded on top of the fibroblasts at a concentration of 5 × 10^3^ cells/well and cultured at 37 °C, 5% CO_2_, 5% O_2_ for 24 h. The in-vitro cellularized co-cultures were then fixed, stained, and imaged for analysis. For experiments involving mast cells, conditioned media was prepared by suspending mast cells (HMC-1 or HMC-1-SAMD14+ cells) in IMDM (Gibco) supplemented with 2% FBS (Gibco) at a concentration of 3 × 10^5^ cells/mL for 48 h. The supernatant from this culture was then collected and filtered with a 0.22 μm filter. BPH-1 cells were suspended in a 1:1 (*v*/*v*) mix of HMC-1 or HMC-1-SAMD14 conditioned media and fibroblast media and seeded. After 24 h, co-cultures were fixed with 4% PFA (Sigma-Aldrich) and permeabilized with 0.1% Triton-X 100 (BDH). After a 30-min blocking step with 1% bovine serum albumin (BSA; Sigma-Aldrich), the co-cultures were incubated with 1:200 dilution of mouse anti-human fibronectin (DSHB, University of Iowa) in PBS for 1 h at room temperature. After primary antibody incubation, the co-culture was incubated with a cocktail mix of anti-mouse Alexa Fluor 647 (1:400 dilution; Invitrogen), rhodamine phalloidin (1:300 dilution; Invitrogen), and 1:1000 dilution of 4,6-diamidino-2-phe-nylindole (DAPI) (1:1000 dilution; Invitrogen) for 30 min at room temperature. After incubation, the cellularized co-culture is washed with phosphate-buffered saline.

The cellularized co-culture were imaged at 405 nm (DAPI- blue), 488 nm (CtG BPH-1 cells—Green), 561 (F-actin—red) and/or 633 nm (fibronectin—far red) using a Nikon C1 Inverted Eclipse 90i confocal microscope equipped with 10× objective lens. All Leica microscopes ran with LAS AF software (Leica MicroSystems) and all Nikon microscopes ran with NIS Elements Software (Nikon).

### 4.15. ECM Orientation Analysis

The distribution of orientation of the ECM fibers was analyzed with ImageJ (NIH) using the Orientation J plugin to generate a pseudocolor visual representation and fiber orientation distributions as previously described [45]. Briefly, the distribution of orientation angles of the ECM fibers was calculated using the orientation and isotropic properties of a region of interest in an image by calculating the individual pixels that made up the ECM fibers. The calculated angle of ECM fibers is represented by a hue-saturation-brightness (HSB) color-coded images, where the different colors correspond to different orientation angle distribution. To quantitatively measure the distribution and orientation of the ECM fibers, a Gaussian window of σ = 2 was applied on the region of interest and the software will compute the value of orientation and gives a quantitative data for the frequency and distribution of angles from −90° to 90°. After normalization of the orientation peak distributions, plots were subjected to a Kruskal–Wallis test with Dunn’s post-hoc multiple comparisons test to determine statistical significance.

### 4.16. BPH-1 Morphology Analysis

2D quantitative analysis of BPH-1 cell morphology was performed using ImageJ (NIH) as previously described [13]. Briefly, to quantitatively determine the morphology of BPH-1 cells after co-culture, all BPH-1 cell with an extension over 5 μm were computed using ImageJ. A maximum intensity projection was applied to the analyzed image, followed by Gaussian Blur filter of σ: 2 and a thresholding and watershed step to obtain a calculated value of the cell’s shape factor, area, cell length, and orientation. To determine orientation of the cell, the standard deviation (StDev) of the cell orientation was applied to the calculated orientation value. Statistics were performed using two-way ANOVA with Tukey’s post hoc test to determine the shape factor, area, and cell length and an unpaired *t*-test or one-way ANOVA with Tukey’s post hoc test for standard deviation of orientation.

### 4.17. Statistical Analysis

All data analysis was conducted using GraphPad Prism 8 software (GraphPad Software Inc.). All data were expressed as mean ± standard error of the mean (SEM) unless otherwise stated. All statistical significance was set to *p* < 0.05 unless specified.

## 5. Conclusions

Mast cells are multifunctional immune cells that reside in the prostate tumor microenvironment and are associated with poor patient outcomes. However, the functional ability of resident mast cells localized within tumor and non-tumor regions of the prostate gland remains unclear. Tumors rely on the bidirectional communication between resident cells and the ECM to create a microenvironment that promotes tumorigenesis and metastasis. Our data present the first profile of human mast cells from prostate cancer specimens and identify novel mast cell-derived SAMD14 as an important mediator of intercellular communication to direct matrix organization and epithelial interaction within the prostate tumor–stromal microenvironment. The identification of distinct mast cell phenotypes and functions may help unveil the complex interactions between mast cells and the tumor microenvironment and provide insights into the regulation and promotion of prostate cancer pathogenesis. Heterotypic interactions between MC–CAF–prostate tumor cells contribute to our understanding of prostate cancer progression and aid in the discovery of adjunct therapeutic targets within the prostate tumor microenvironment.

## Data Availability

The data presented in this study are available in this article and the Supplementary Materials. In addition, the mass spectrometry proteomics data in this study have been deposited to the ProteomeXchange Consortium via the PRIDE partner repository with the dataset identifier PXD022782.

## Supplementary Materials

The following are available online at https://www.mdpi.com/2072-6694/13/6/1237/s1. Figure S1: Validation of non-tumor and tumor regions in human prostate tissue. (a) Macroscopic dissection of non-malignant (blue circle) and tumor (red circle) regions of radical prostatectomy tissue was determined by a trained pathologist. (b) Immunohistochemistry images shows human prostate tissue stained with AMACR+ (pink) and p63+ (brown) in matched non-tumor and tumor prostate tissues. Scale bars = 200 µm. Images shown are representative (n = 5). Figure S2: Expression of SAMD14+ tryptase+ mast cells in human prostate tissue section. (a) Images show human prostate tissue stained with SAMD14+ (brown) and tryptase+ mast cells (red) in matched non-tumor and tumor prostate tissues from 3 patients with corresponding isotype control. Scale bars = 100 µm. Images shown per patient are representative (n = 3). (b) The percentage of tryptase+ mast cells positive for low intensity (+1; yellow), moderate intensity (+2; orange) and high intensity (+3; red) SAMD14 staining in the 3 patients. Figure S3: (a) Growth curves show in vitro cultures of primary mast cells isolated from tumor (MC-T) and non-tumor (MC-NT) prostate regions from two separate patients (P359 and P371). No growth was observed over 100 days in culture. (b) Growth Kinetics for HMC1 and HMC-1-SAMD14+ cell lines over 7 days in culture. Each point represents the average viable count for triplicate wells +/− SEM. Figure S4: Raw western blot images of SAMD14 and β-actin protein expression in HMC-1 and HMC-1-SAMD14+ cell line. 25 µg of protein was loaded per lane. Proteins collected for HMC-1 and HMC-1-SAMD14+ cell-lines were repeated 3 times. Figure S5: SAMD14 reduction in mast cells converts normal NPF-ECM to a tumorigenic CAF-like ECM and promotes a pro-tumor epithelial morphology (a) Representative images show BPH-1 cell morphology when cultured with fibroblasts (CAF/NPF) derived from P128. Corresponding fibronectin staining after image processing to represent the degree of ECM fiber alignment produced by (i) NPF, (ii) CAF and (iii) NPF + HMC-1 CM and (iv) NPF + HMC-1-SAMD14+ CM. Scale bar: 100µm. (b) Quantification of fiber alignment for NPF, CAF and NPF cultured with HMC-1 CM and HMC-1-SAMD14+ CM for the CAF and NPF derived from P128 and P332. Line plots represent analysis with 4 technical replicates per patient, from 4 images per replicate. Statistics performed using Kruskal–Wallis test with Dunn’s post-hoc multiple comparisons test (*, *p* <0.01) to determine statistical significance. Data represented as mean. (c) Quantification of (i) shape factor, (ii) area, (iii) cell length and (iv) standard deviation of orientation of BPH-1 cells cultured on NPF, CAF, NPF + HMC-1 CM and NPF + HMC-1 SAMD14+ CM for the CAF and NPF derived from P128 and P332. Box and whisker plots represent the max to min value of BPH-1 shape factor, area, cell length and standard deviation of orientation. Graphs represent analysis of 3 images per replicate and 4 replicates are conducted per patient (>50 BPH-1 cells/image). Statistics performed using two-way ANOVA with Tukey’s post hoc test for average shape factor, area and cell length (* = *p* < 0.01 compared to NPF; # = *p* < 0.05 compared to CAF; ^ = *p* < 0.01 compared to CAF+ HMC-1) and a one-Way ANOVA with Tukey’s post hoc for average standard deviation of orientation (* = *p* < 0.05 compared to NPF; # = *p* < 0.05 compared to CAF; ^ = *p* < 0.05 compared to CAF+ HMC-1). Figure S6: HMC-1 and HMC-1-SAMD14+ conditioned media (CM) has no direct effect on BPH-1 morphology. (a) Representative image of fluorescent-labelled BPH-1 cells cultured with control, HMC-1 or HMC-1-SAMD14+ CM for 24 h. (b) BPH-1 morphology based on (i) shape factor, (ii) area, (iii) cell length and (iv) standard deviation of orientation was quantified using Image J software. Box and whisker plots represent the max to min value of BPH-1 shape factor, area, cell length and standard deviation of orientation. Images are representative (n = 6). Scale Bar = 100 µM. Figure S7: SAMD14 mast cell affects the CAF matrix alignment causing changes to the prostate epithelial phenotype (a) Representative image of CAF and NPF derived from P128. BPH-1 cell morphology and their corresponding fibronectin staining showing ECM fiber alignment for cell-derived matrices produced by (i) NPF, (ii) CAF and (iii) CAF + HMC-1 CM and (iv) CAF + HMC-1-SAMD14+ CM. ECM images were processed and color-coded to represent the degree of fiber orientation distribution within each sample. Scale bar: 100 µm. (b) Quantification of fiber alignment for NPF, CAF and CAF cultured with HMC-1 CM and HMC-1-SAMD14+ CM for the CAF and NPF derived from P128 and P332. Line plots represent analysis with 4 technical replicates per patient, from 4 images per replicate. Statistics performed using Kruskal–Wallis test with Dunn’s post-hoc multiple comparisons test (*, *p* < 0.001) to determine statistical significance. Data represented as mean. (c) Quantification of BPH-1 morphology based on (i) shape factor, (ii) area, (iii) cell length and (iv) standard deviation of orientation after cultured on NPF, CAF, CAF + HMC-1 CM and CAF + HMC-1 SAMD14+ CM for the CAF and NPF derived from P128 and P332. Box and whisker plots represent the max to min. Graphs represent analysis of 3 images per replicate and 4 replicates are conducted per patient (>50 BPH-1 cells/image). Statistics performed using two-way ANOVA with Tukey’s post hoc test for average shape factor, area and cell length (* = *p* < 0.001 compared to NPF; # = *p* < 0.001 compared to CAF; ^ = *p* < 0.001 compared to CAF+ HMC-1) and a one-way ANOVA with Tukey’s post hoc for average standard deviation of orientation (* = *p* < 0.01 compared to NPF; # = *p* < 0.01 compared to CAF; ^ = *p* < 0.01 compared to CAF+ HMC-1). Supplementary material and methods. Table S1: Clinical features and follow-up of patient radical prostatectomy tissue for the discovery and validation cohort. Table S2: Mast cells percentage and viable cell numbers isolated from primary human prostate tumor and non-tumor tissue for the discovery cohort. Table S3: Gene set enrichment analysis results with hallmark gene sets on primary mast cells RNAseq dataset. Whole genes ordered by z-score from the meta-analysis were used as ranked gene for analysis. Enriched biological pathways in MC-T relative to MC-NT; Normalized enrichment score (NES). Table S4: Differentially expressed genes in MC-T dataset compared to MC-NT dataset (FDR < 0.1 and FC > 2). Table S5: Total secreted proteins identified from HMC-1 and HMC-1-SAMD14 conditioned media. Table S6: Secreted proteins enriched in HMC-1 SAMD14+ conditioned media compared to HMC1 conditioned media based on a threshold of FC > 2 and *p* value < 0.05. Table S7: Gene Ontology enrichment analysis of the differentially secreted proteins enriched in HMC-1-SAMD14+ CM compared to HMC-1 CM (*p* < 0.05; FC > 2). Table S8: Gene Ontology enrichment analysis of the differentially secreted proteins reduced in HMC-1-SAMD14+ CM compared to HMC-1 CM (*p* < 0.05; FC < −1.5). Table S9: Antibody details and staining conditions for immunohistochemistry. Table S10: Clinicopathological features of tumors of origin for cultured fibroblast cell-lines (Cancer associated fibroblasts—CAFs and Non-malignant prostate fibroblasts—NPFs). Primary patient-matched prostatic fibroblasts were derived from a varied cohort of men. Cohort was varied in terms of age, focality and invasion and represents moderate to high localized prostate cancer (GG3 and GG5). n/a—information not available.
