# Supplementary material for: Mast Cell-Derived SAMD14 Is a Novel Regulator of the Human Prostate Tumor Microenvironment
Source: Cancers (Basel). 2021 Mar 11;13(6):1237. doi: 10.3390/cancers13061237 (PMC7999778; doi:10.3390/cancers13061237)
Supplement: Supplementary file 1 [file cancers-13-01237-s001.zip › Cancers_1133487_Supplementary Material and Methods.docx]

Supplementary Material and Methods

1.1. In-vitro cellularized ‘washout assay’ co-culture assay

For ‘washout assay’, an *in-vitro* cellularized co-culture model was used as described in section 4.14. Briefly, patient-matched primary CAFs or NPFs were seeded in 48 well plates at a concentration of 1.0 X 10^4^ cells/mL and cultured for 8–10 days in fibroblast media to yield a dense monolayer of extensive ECM deposition. HMC-1 or HMC-1-SAMD14+ CM was added to the CAF cultures for 24h. After 24h, CM was washed off with fresh fibroblast media prior to the seeding of BPH-1 cells, pre-stained with CellTracker Green CMFDA (CtC; Invitrogen), on top of the fibroblasts at a concentration of 5 X 10^3^ cells/well. The co-culture was incubated at 37°C, 5% CO_2_, 5% O_2_ for 24h. After 24h, co-cultures were fixed with 4% PFA (Sigma-Aldrich) and permeabilized with 0.1% Triton-X 100 (BDH). After a 30-minute blocking step with 1% bovine serum albumin (BSA; Sigma-Aldrich), the co-cultures were incubated with 1:200 dilution of mouse anti-human ﬁbronectin (DSHB, University of Iowa) in PBS for 1h at room temperature. After primary antibody incubation, the co-culture was incubated with a cocktail mix of anti-mouse Alexa Fluor 647 (1:400 dilution; Invitrogen), rhodamine phalloidin (1:300 dilution; Invitrogen), and 1:1000 dilution of 4,6-diamidino-2-phe-nylindole (DAPI) (1:1000 dilution; Invitrogen) for 30 min at room temperature before being washed and stored in phosphate-buffered saline. The cellularized co-culture were imaged at 405 nm (DAPI- blue), 488 nm (CtG BPH-1 cells - Green), 561 (F-actin - red) and/or 633 nm (ﬁbronectin – far red) using a Nikon C1 Inverted Eclipse 90i confocal microscope equipped with 10× objective lens. All Nikon microscopes ran with NIS Elements Software (Nikon). See section 4.15 for ECM orientation analysis and section 4.16 for BPH-1 morphology analysis.

1.2. BPH-1 morpholgy after treatment with HMC-1 and HMC-1-SAMD14+ CM

BPH-1 cells pre-stained with CellTracker Green CMFDA (CtC; Invitrogen) were treated with Mitomycin C (Sigma-Aldrich) at a concentration of 20µg/ml for 2h. Cells were washed twice with fresh fibroblast media before seeding in a 24 well plate at a concentration of 5 X 10^4^ cells/ml in fibroblast media, HMC-1 CM or HMC-1-SAMD14+ CM. The culture was incubated at 37°C, 5% CO_2_, 5% O_2_ for 24h. The BPH-1 culture was then fixed with 4% PFA (Sigma-Aldrich) and imaged at 488nm using a Nikon C1 Inverted Eclipse 90i confocal microscope. See section 4.16 for BPH-1 morphology analysis.
