# Supplementary material for: Mast Cell-Derived SAMD14 Is a Novel Regulator of the Human Prostate Tumor Microenvironment
Source: Cancers (Basel). 2021 Mar 11;13(6):1237. doi: 10.3390/cancers13061237 (PMC7999778; doi:10.3390/cancers13061237)
Supplement: Supplementary file 1 [file cancers-13-01237-s001.zip › cancers-1133487supplpublish (1).pdf]

Supplementary Materials: Mast Cell-Derived SAMD14 is a Novel Regulator of the Human Prostate Tumor Microenvironment

Linda K. H. Teng, Brooke A. Pereira, Shivakumar Keerthikumar, Cheng Huang, Birunthi Niranjan, Sophie N. Lee, Michelle Richards, Ralf B. Schittenhelm, Luc Furic, David L. Goode, Mitchell G. Lawrence, Renea A. Taylor, Stuart J. Ellem, Gail P. Risbridger and Natalie L. Lister

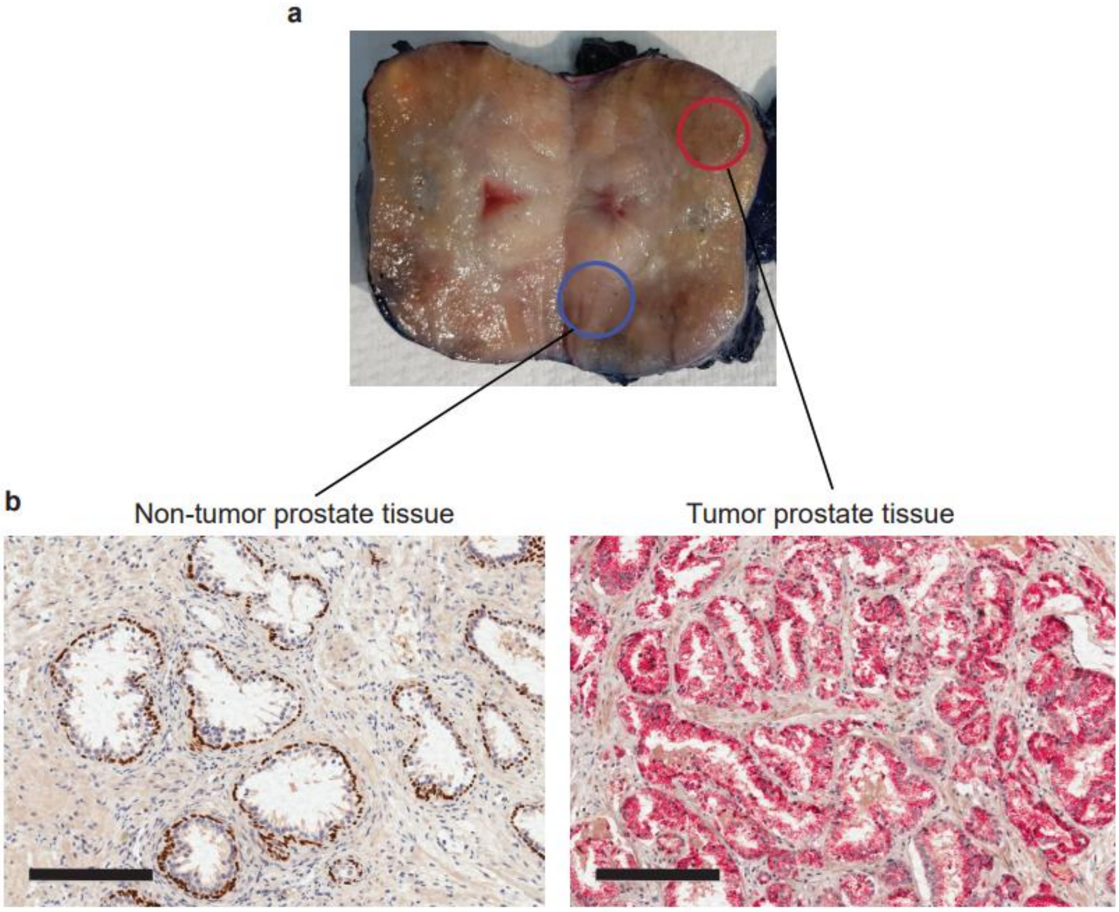

Figure S1. Validation of non-tumor and tumor regions in human prostate tissue. (a) Macroscopic dissection of non-malignant (blue circle) and tumor (red circle) regions of radical prostatectomy tissue was determined by a trained pathologist. (b) Immunohistochemistry images shows human prostate tissue stained with AMACR+ (pink) and p63+ (brown) in matched non-tumor and tumor prostate tissues. Scale bars = 200 µm. Images shown are representative (n = 5).

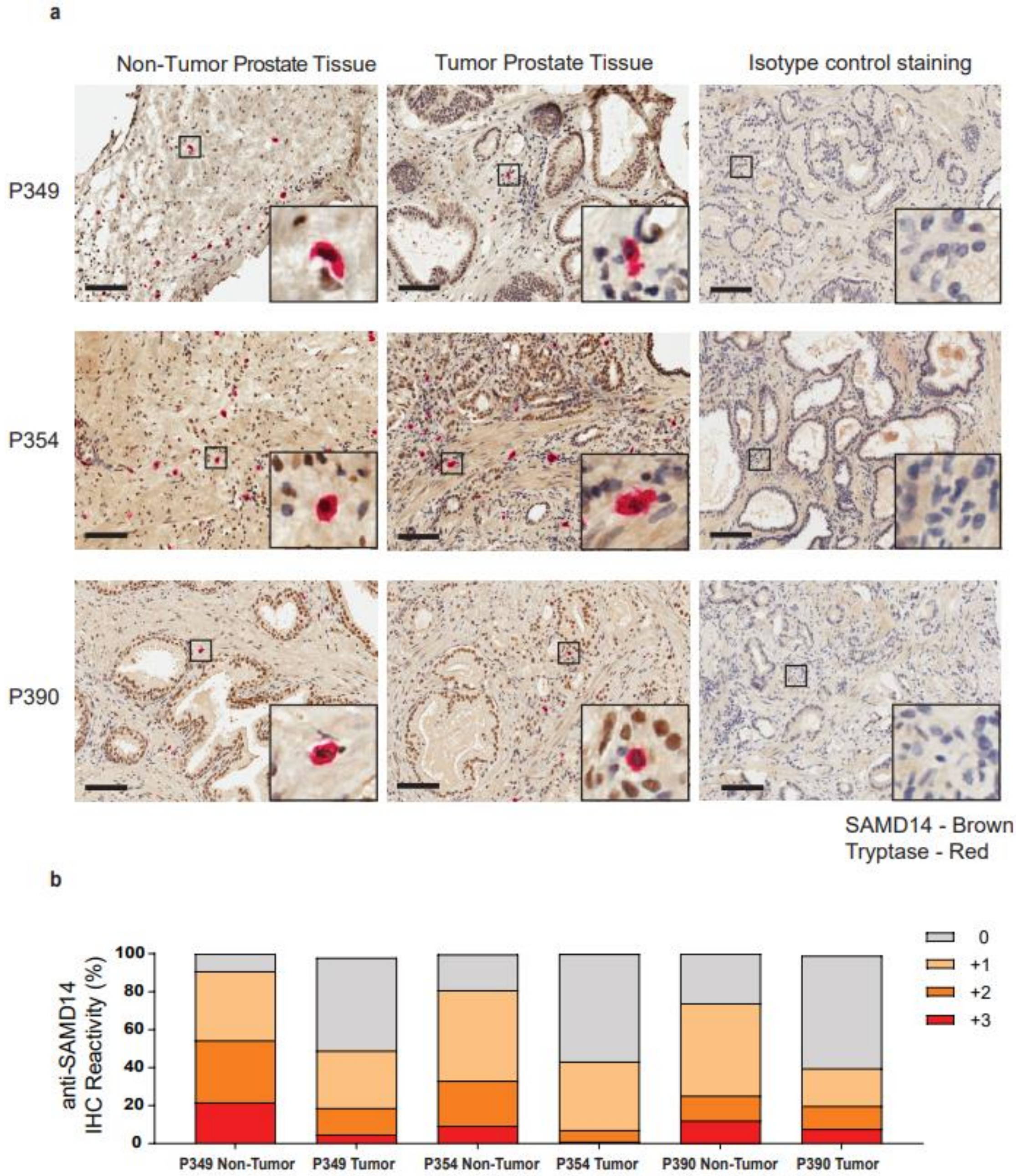

Figure S2. Expression of SAMD14+ tryptase+ mast cells in human prostate tissue section. (a) Images show human prostate tissue stained with SAMD14+ (brown) and tryptase+ mast cells (red) in matched non-tumor and tumor prostate tissues from 3 patients with corresponding isotype control. Scale bars = 100 µm. Images shown per patient are representative (n = 3). (b) The percentage of tryptase+ mast cells positive for low intensity (+1; yellow), moderate intensity (+2; orange) and high intensity (+3; red) SAMD14 staining in the 3 patients.

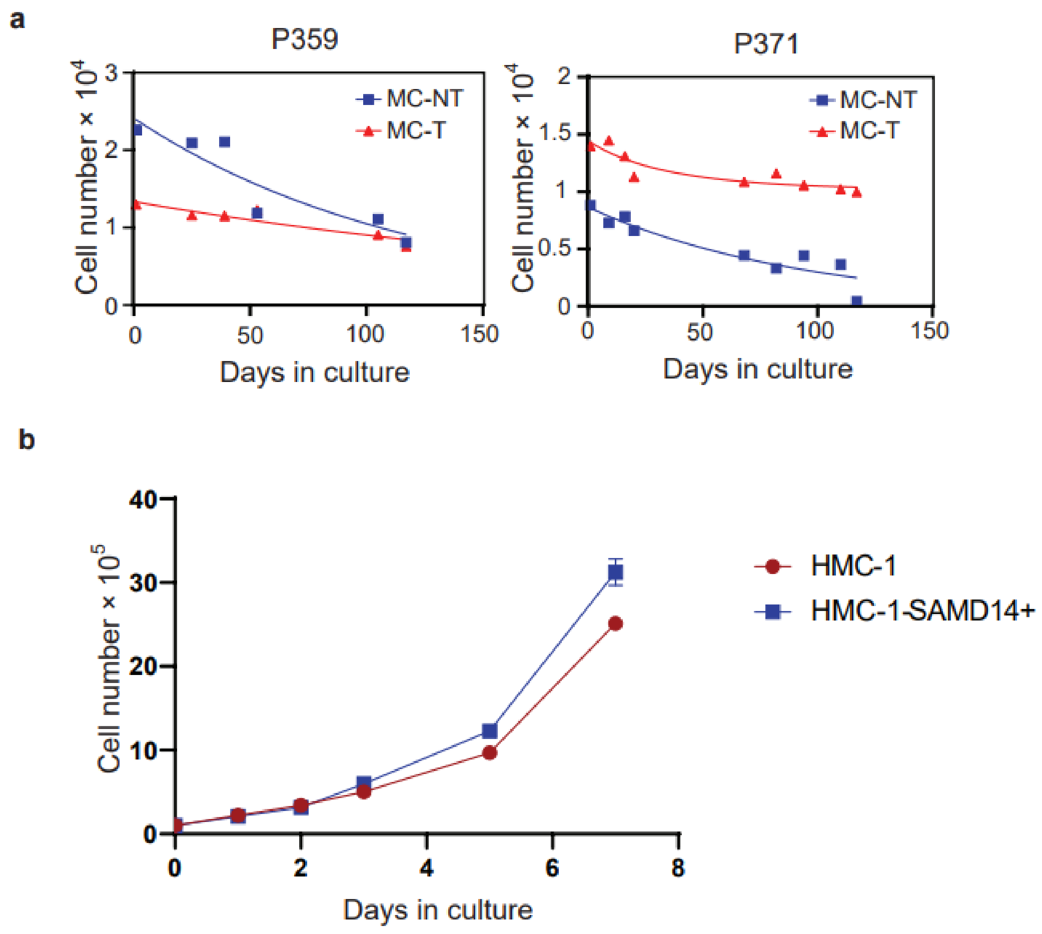

Figure S3. (a) Growth curves show in vitro cultures of primary mast cells isolated from tumor (MC-T) and non-tumor (MC-NT) prostate regions from two separate patients (P359 and P371). No growth was observed over 100 days in culture. (b) Growth Kinetics for HMC1 and HMC-1-SAMD14+ cell lines over 7 days in culture. Each point represents the average viable count for triplicate wells +/- SEM.

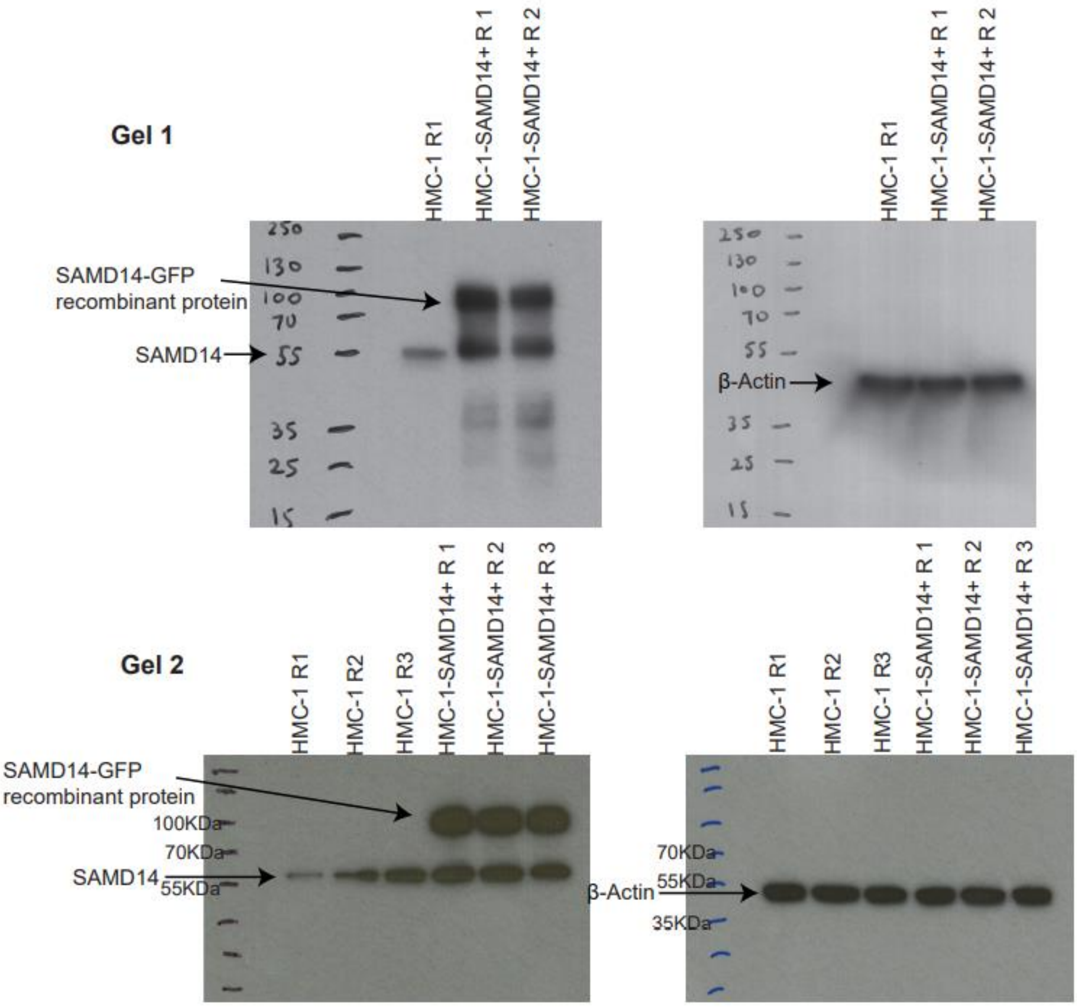

Figure S4. Raw western blot images of SAMD14 and β-actin protein expression in HMC-1 and HMC-1-SAMD14+ cell line. 25 μg of protein was loaded per lane. Proteins collected for HMC-1 and HMC-1-SAMD14+ cell-lines were repeated 3 times.

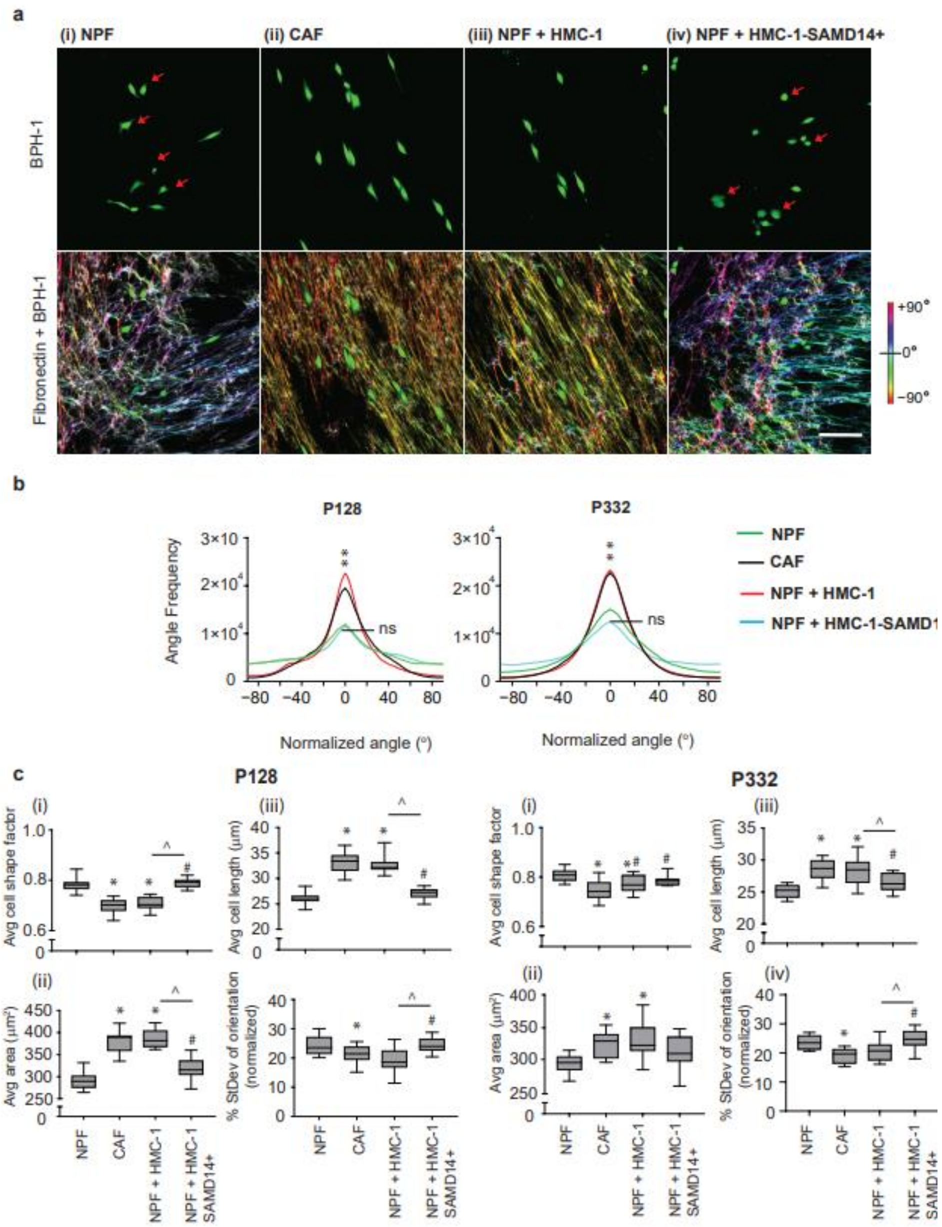

Figure S5. SAMD14 reduction in mast cells converts normal NPF-ECM to a tumorigenic CAF-like ECM and promotes a pro-tumor epithelial morphology (a) Representative images show BPH-1 cell morphology when cultured with fibroblasts (CAF/NPF) derived from P128. Corresponding fibronectin staining after image processing to represent the degree of ECM fiber alignment produced by (i) NPF, (ii) CAF and (iii) NPF + HMC-1 CM and (iv) NPF + HMC-1-SAMD14+ CM. Scale bar: 100µm. (b) Quantification of fiber alignment for NPF, CAF and NPF cultured with HMC-1 CM and HMC-1-SAMD14+ CM for the CAF and NPF derived from P128 and P332. Line plots represent analysis with 4 technical replicates per patient, from 4 images per replicate. Statistics performed using Kruskal–Wallis test with Dunn's post-hoc multiple comparisons test (*, p <0.01) to determine statistical significance. Data represented as mean. (c) Quantification of (i) shape factor, (ii) area, (iii) cell length and (iv) standard deviation of orientation of BPH-1 cells cultured on NPF, CAF, NPF + HMC-1 CM and NPF + HMC-1 SAMD14+ CM for the CAF and NPF derived from P128 and P332. Box and whisker plots represent the max to min value of BPH-1 shape factor, area, cell length and standard deviation of orientation. Graphs represent analysis of 3 images per replicate and 4 replicates are conducted per patient (>50 BPH-1 cells/image). Statistics performed using two-way ANOVA with Tukey's post hoc test for average shape factor, area and cell length (* = p < 0.01 compared to NPF; # = p < 0.05 compared to CAF; ^ = p < 0.01 compared to CAF+ HMC-1) and a one-Way ANOVA with Tukey's post hoc for average standard deviation of orientation (* = p < 0.05 compared to NPF; # = p < 0.05 compared to CAF; ^ = p < 0.05 compared to CAF+ HMC-1.

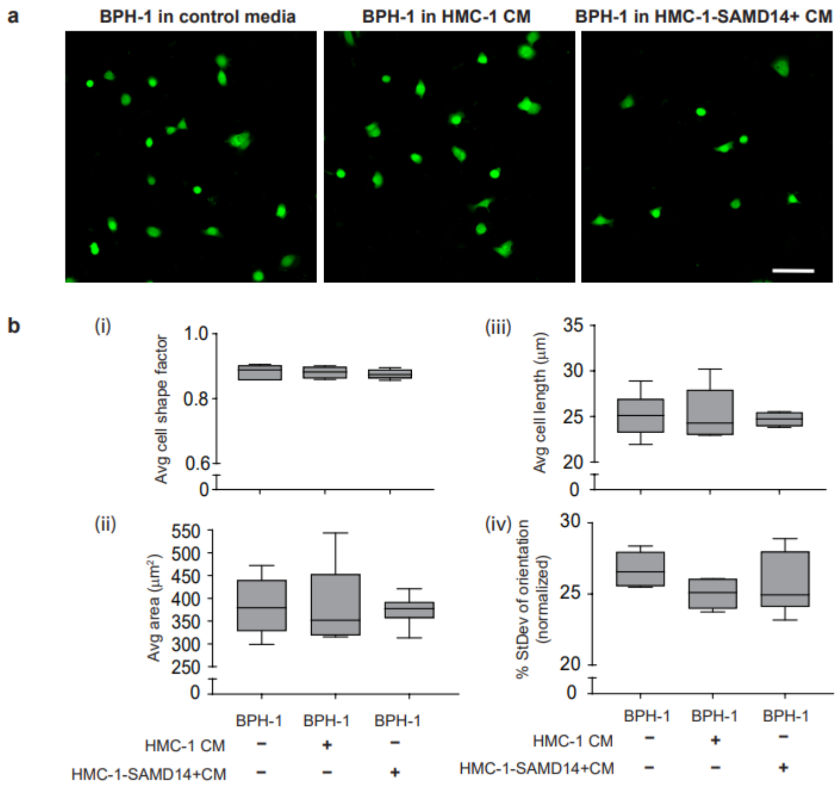

Figure S6. HMC-1 and HMC-1-SAMD14+ conditioned media (CM) has no direct effect on BPH-1 morphology. (a) Representative image of fluorescent-labelled BPH-1 cells cultured with control, HMC-1 or HMC-1-SAMD14+ CM for 24 h. (b) BPH-1 morphology based on (i) shape factor, (ii) area, (iii) cell length and (iv) standard deviation of orientation was quantified using Image J software. Box and whisker plots represent the max to min value of BPH-1 shape factor, area, cell length and standard deviation of orientation. Images are representative (n = 6). Scale Bar = 100 µM.

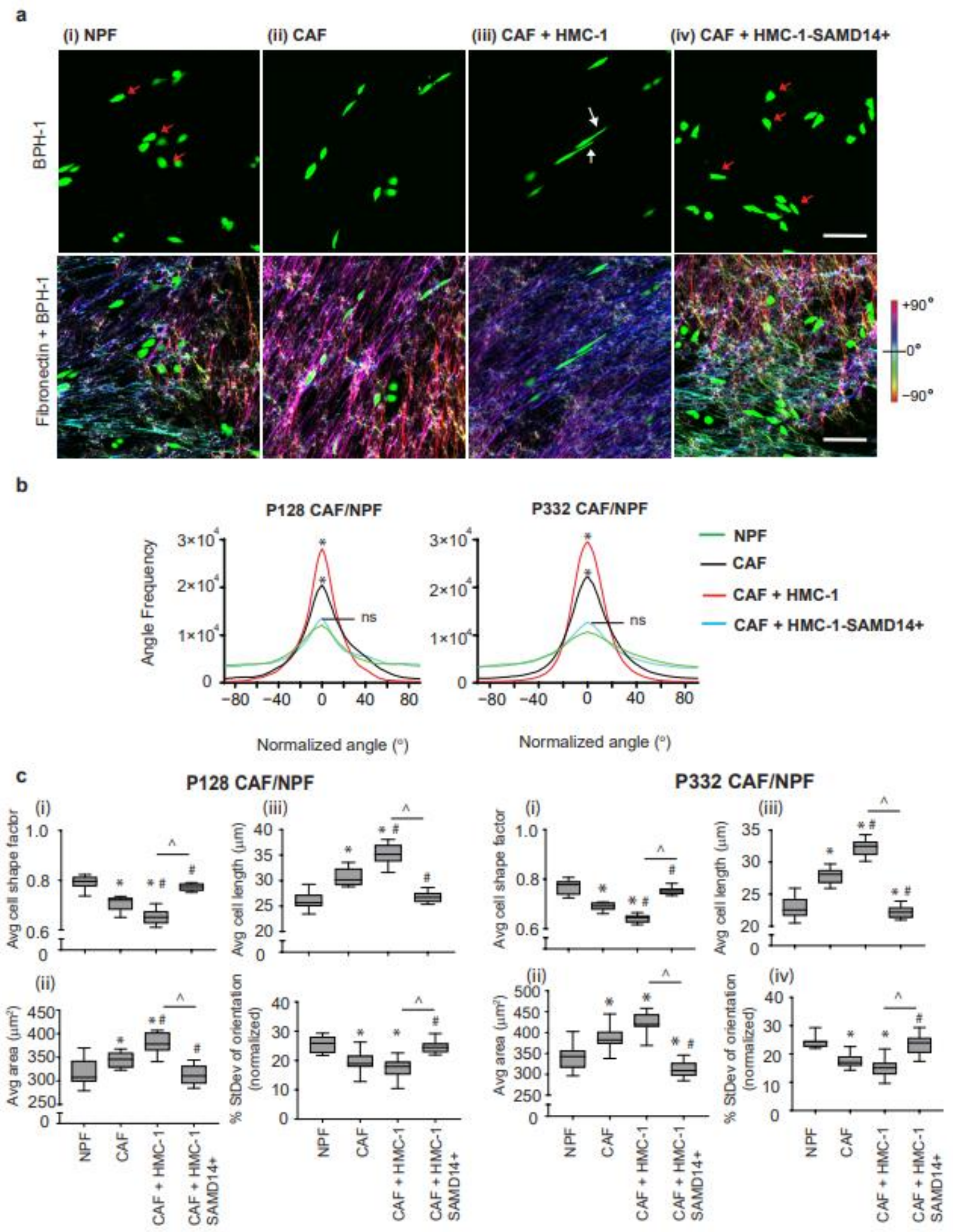

Figure S7. SAMD14 mast cell affects the CAF matrix alignment causing changes to the prostate epithelial phenotype (a) Representative image of CAF and NPF derived from P128. BPH-1 cell morphology and their corresponding fibronectin staining showing ECM fiber alignment for cell-derived matrices produced by (i) NPF, (ii) CAF and (iii) CAF + HMC-1 CM and (iv) CAF + HMC-1-SAMD14+ CM. ECM images were processed and color-coded to represent the degree of fiber orientation distribution within each sample. Scale bar: 100 µm. (b) Quantification of fiber alignment for NPF, CAF and CAF cultured with HMC-1 CM and HMC-1-SAMD14+ CM for the CAF and NPF derived from P128 and P332. Line plots represent analysis with 4 technical replicates per patient, from 4 images per replicate. Statistics performed using Kruskal–Wallis test with Dunn's post-hoc multiple comparisons test (*, $p < 0.001$) to determine statistical significance. Data represented as mean. (c) Quantification of BPH-1 morphology based on (i) shape factor, (ii) area, (iii) cell length and (iv) standard deviation of orientation after cultured on NPF, CAF, CAF + HMC-1 CM and CAF + HMC-1 SAMD14+ CM for the CAF and NPF derived from P128 and P332. Box and whisker plots represent the max to min. Graphs represent analysis of 3 images per replicate and 4 replicates are conducted per patient (>50 BPH-1 cells/image). Statistics performed using two-way ANOVA with Tukey's post hoc test for average shape factor, area and cell length (* = $p < 0.001$ compared to NPF; # = $p < 0.001$ compared to CAF; ^ = $p < 0.001$ compared to CAF+ HMC-1) and a one-way ANOVA with Tukey's post hoc for average standard deviation of orientation (* = $p < 0.01$ compared to NPF; # = $p < 0.01$ compared to CAF; ^ = $p < 0.01$ compared to CAF+ HMC-1). Supplementary material and methods.
